# The database of chromosome imbalance regions and genes resided in lung cancer from Asian and Caucasian identified by array-comparative genomic hybridization

**DOI:** 10.1186/1471-2407-12-235

**Published:** 2012-06-12

**Authors:** Fang-Yi Lo, Jer-Wei Chang, I-Shou Chang, Yann-Jang Chen, Han-Shui Hsu, Shiu-Feng Kathy Huang, Fang-Yu Tsai, Shih Sheng Jiang, Rajani Kanteti, Suvobroto Nandi, Ravi Salgia, Yi-Ching Wang

**Affiliations:** 1 Department of Pharmacology and Institute of Basic Medical Sciences, College of Medicine, National Cheng Kung University, No.1, University Road, Tainan 701, Taiwan; 2 Institute of Cancer Research, National Health Research Institutes, Miaoli, Taiwan; 3 Department of Life Sciences and Institute of Genome Sciences, National Yang Ming University, Taipei, Taiwan; 4 Institute of Emergency and Critical Care Medicine, National Yang Ming University and Division of Thoracic Surgery, Taipei Veterans General Hospital, Taipei, Taiwan; 5 Division of Molecular and Genomic Medicine, National Health Research Institutes, Miaoli, Taiwan; 6 Department of Medicine, Cancer Research Center, The University of Chicago Medical Center, Pritzker School of Medicine, Chicago, IL, USA

**Keywords:** Array-CGH, Lung cancer, Asian, Caucasian, Oncogene

## Abstract

**Background:**

Cancer-related genes show racial differences. Therefore, identification and characterization of DNA copy number alteration regions in different racial groups helps to dissect the mechanism of tumorigenesis.

**Methods:**

Array-comparative genomic hybridization (array-CGH) was analyzed for DNA copy number profile in 40 Asian and 20 Caucasian lung cancer patients. Three methods including MetaCore analysis for disease and pathway correlations, concordance analysis between array-CGH database and the expression array database, and literature search for copy number variation genes were performed to select novel lung cancer candidate genes. Four candidate oncogenes were validated for DNA copy number and mRNA and protein expression by quantitative polymerase chain reaction (qPCR), chromogenic *in situ* hybridization (CISH), reverse transcriptase-qPCR (RT-qPCR), and immunohistochemistry (IHC) in more patients.

**Results:**

We identified 20 chromosomal imbalance regions harboring 459 genes for Caucasian and 17 regions containing 476 genes for Asian lung cancer patients. Seven common chromosomal imbalance regions harboring 117 genes, included gain on 3p13-14, 6p22.1, 9q21.13, 13q14.1, and 17p13.3; and loss on 3p22.2-22.3 and 13q13.3 were found both in Asian and Caucasian patients. Gene validation for four genes including *ARHGAP19* (10q24.1) functioning in Rho activity control, *FRAT2* (10q24.1) involved in Wnt signaling, *PAFAH1B1* (17p13.3) functioning in motility control, and *ZNF322A* (6p22.1) involved in MAPK signaling was performed using qPCR and RT-qPCR. Mean gene dosage and mRNA expression level of the four candidate genes in tumor tissues were significantly higher than the corresponding normal tissues (*P*<0.001~*P*=0.06). In addition, CISH analysis of patients indicated that copy number amplification indeed occurred for *ARHGAP19* and *ZNF322A* genes in lung cancer patients. IHC analysis of paraffin blocks from Asian Caucasian patients demonstrated that the frequency of PAFAH1B1 protein overexpression was 68% in Asian and 70% in Caucasian.

**Conclusions:**

Our study provides an invaluable database revealing common and differential imbalance regions at specific chromosomes among Asian and Caucasian lung cancer patients. Four validation methods confirmed our database, which would help in further studies on the mechanism of lung tumorigenesis.

## Background

Lung cancer is the leading cause of cancer death worldwide [1,2]. There is mounting evidence of difference between Asian and Caucasian lung cancer patients in clinical and epidemiological aspects. For example, Lee *et al*. [3] reported that lung cancer in Taiwan and South China is characterized with a high percentage of female adenocarcinoma (ADC) and nonsmokers. In addition, response to certain anticancer treatments such as gefitinib is much greater in Asian patients than Caucasians [4]. However, these are rare large-scale attempts to identify the molecular characteristics of lung cancer between Asian and Caucasian. Therefore, identification and characterization of the chromosome regions with DNA copy number alteration in Western and Asian countries is important to unravel the mechanism underlying lung tumorigenesis.

Genomic DNA copy number variation is a hallmark of cancer and can lead to alteration in the expression and functions of genes residing within the affected chromosomal region [5]. Such a segment in the tumor genome may harbor either oncogenes or tumor suppressor genes depending on whether they are present in increased or decreased copy number, respectively. Identification of regions with copy number aberrations and the genes involved thus offers a basis for better understanding of cancer development [6]. Array-comparative genomic hybridization (array-CGH) can measure relative copy number at specific loci, which are represented by arrays of mapped bacterial artificial chromosomes (BAC) clones, cDNA clones, or various oligonucleotide-based formats [7-9]. The resolution of array-CGH is determined by the genomic spacing of the array elements thus is much more improved compared to previous conventional CGH [10-12].

To identify the chromosome regions with DNA copy number alteration in patients from Western and Asian countries, we constructed a non-gapped array containing 3604 BAC clones covering 18 lung cancer-related chromosome imbalance hotspot regions identified in our previous study [13]. DNA from 40 Asian and 20 Caucasian non-small cell lung cancer (NSCLC) patients was analyzed using this specialized array to identify lung cancer-related genes either common or differential in these two ethnic groups. The Bayes statistical and comparative genomic approaches were used to identify genes with DNA copy number alterations. Our study provides a comprehensive database for chromosomal regions and genes with copy number imbalance in both Asian and Caucasian lung cancer patients. In addition, gene validation was performed using quantitative polymerase chain reaction (qPCR), reverse transcriptase-qPCR (RT-qPCR), chromogenic *in situ* hybridization (CISH), and immunohistochemistry (IHC) on specimens from the Asian and Caucasian lung cancer patients.

## Methods

### Clinical samples preparation and DNA/RNA extraction

Tissues were collected after obtaining appropriate institutional review board permission (VGHIRB No. 201001019IA) and informed consent from the recruited patients. Surgically resected tumor tissue and corresponding normal tissue were collected from 40 patients diagnosed with primary non-small cell lung cancer (NSCLC) admitted to Taipei Veterans General Hospital, Taiwan. Twenty NSCLC tumor tissues from Caucasian patients were obtained from the University of Chicago, USA. The list of these 60 patients along with the patients used for further validations is described in the Additional file 1: Table S1. Histological classification was determined according to the WHO classification system and the tumor-node-metastasis system. Information on the age, sex, tumor type, tumor stage and smoking history of the patients was obtained from hospital records. The genomic DNA was prepared using proteinase K digestion and phenol-chloroform extraction. Total RNA was prepared from tumors and normal lung tissues, using TRIzol reagent (Invitrogen, Carlsbad, CA). cDNA was generated using SuperScript reverse transcriptase (Invitrogen).

### Array-CGH

The genomic microarray used contained 3604 BAC clones representing the human genome at 18 non-gap chromosome regions ( Additional file 2: Table S2). Clone mapping was analyzed using the BAC end pairs database of UCSC Genome browser (http://genome.ucsc.edu/, build 36 - hg18). The BAC library used was RP-11. The extracted BAC DNA was amplified to 10 μg using Phi29 DNA polymerase reaction (TempliPhi DNA Sequencing Template Amplification Kit, Amersham, Princeton, NJ), then digested with DNaseI (New England Biolabs, Ipswich, MA), and purified with MultiScreen-PCR Plates (Millipore, Danvers, MA). After digestion, the DNA fragments were resolved by gel electrophoresis and the fragment sizes were optimized to within 0.5–3 kb. Finally, the purified BAC DNA fragments were spotted in triplicate of 72 blocks onto the glass slides (Corning, NY) with spotting solution (50% DMSO, 50% dH_2_O). Representative array images are shown in the Additional file 3: Figure S1.

Approximately 500 *n*g of tumor genomic DNA and reference DNA [for Asian samples: 28 matched-normal genomic DNA pool; for Caucasian samples: male human genomic DNA (Promega, Leiden, the Netherlands)] were labeled by random priming using BioPrime DNA Labeling System (Invitrogen) with Cy3-dCTP (reference) or Cy5-dCTP (tumor). Labeled tumor and reference DNA were combined together with 100 μg human Cot-1 DNA (Invitrogen). Hybridization was performed using MAUI hybridization system (BioMcro Systems, Salt Lake City, UT) by agitating the hybridization solution for 50 hours at 50°C. The arrays were scanned using scanner GenePix4000B (Axon Instruments, Foster City, CA) and the images were segmented and transformed to Cy3-Cy5 log ratio using GenePix software (Axon Instruments, Inc). Further data processing, including normalization and data analysis was performed and is described in detail in the Result section.

### Biological software analysis for candidate gene selection

The array results were further analyzed by MetaCore™ analysis software (GeneGo, Inc., St. Joseph, MI) to identify genes involved in cellular pathways, disease-specific biomarker genes, and genes annotated in the gene ontology databases. Calculation of statistical significance throughout MetaCore was based on *P* value, calculated based on hypergeometric distribution [14]. *P* value of 0.05 was used for the cutoff of candidate gene selection.

### Real-time quantitative polymerase chain reaction (qPCR)

qPCR was used to confirm the array-CGH results and to determine the DNA copy number variations of identified candidate genes in 30 NSCLC tumor and corresponding normal samples from Asian, and 30 NSCLC tumor and 12 autopsy samples from Caucasian. DNA copy number was quantified by qPCR using the ABI 7900 Sequence Detection System (PE Applied Biosystems, Foster City, CA). Genomic DNA (100 *n*g) was amplified using the SYBR Green PCR Master Mix (Roche, Palo Alto, CA) and specific primers for each candidate gene located in exon and adjacent intron, and the *GAPDH* internal control gene located within the promoter region. The primer sequences and annealing temperature are listed in the Additional file 4: Table S3. The mean DNA dosage ratio between each candidate gene and the *GAPDH* internal control gene was calculated in all normal and all tumor samples analyzed [15].

### Quantitative reverse transcription polymerase chain reaction (RT-qPCR)

RT-qPCR was used to measure the mRNA expression of four candidate genes in 45 NSCLC tumor and the corresponding normal samples from Asian, and 24 tumor and corresponding normal samples from Caucasian (TissueScan Lung Cancer Panel IV: HLRT304; OriGene, Rockville, MD). RT-qPCR was conducted using the ABI 7900 Sequence Detection System (PE Applied Biosystems). Total RNA (4 μg) from tumor and the corresponding normal tissues were reverse transcribed into cDNA and amplified using the SYBR Green PCR Master Mix (Roche) and specific primers for each candidate gene and the *ß-actin* internal control gene. The primer sequences and annealing temperature are listed in the Additional file 4: Table S3. The mean mRNA expression ratio between each candidate gene and the *ß-actin* internal control gene was calculated in all normal and all tumor samples analyzed.

### Chromogenic *in situ* hybridization (CISH)

CISH was done on 4 μm thick formalin-fixed paraffin embedded tissue section slides following the standard protocol described in Chang *et al*. [16]. The probes were digoxigenin-labeled with BAC DNA (RP11-375N9 and RP11-170O19 for *ARHGAP19* gene probe; RP11-391k6 for *ZNF322A* gene probe), and then ethanol precipitated with 10 μg Human Cot-1 DNA. At least 100 non-overlapping and intact tumor nuclei were evaluated. The tumor was considered as CISH amplification positive if the copy number was ≥4 signals per nucleus in more than 40% of tumor cells evaluated [16].

### Immunohistochemistry (IHC)

The protein expression level of the candidate gene, *PAFAH1B1*, was evaluated by IHC in 28 NSCLC samples from Asian and 37 lung cancer samples from Caucasian tissue array. Paraffin blocks of tumors were cut into 5-μm slices and then processed using standard deparaffinization and rehydration techniques. Polyclonal antibody against PAFAH1B1 (1:800; Novus, Littleton, CO) was used to detect the protein expression. The evaluation of IHC was conducted blindly without prior knowledge of the clinical and pathologic characteristics of the cases. The samples were graded high expression when >50% tumor cells were stained positive using adequate staining in surrounding normal stromal and epithelial cells.

### Statistical analysis

The Bayes regression approach was used to determine the chromosomal breaking points [17]. Candidate gene selection by MetaCore (GeneGo, Inc.) was calculated based on hypergeometric distribution [14] and a *P* value of 0.05 was used for the cutoff. A two-tailed *t* test was used to determine the statistical significance of difference in DNA dosage and mRNA expression level of candidate genes in clinical samples. Chi-square test was conducted to examine the association between overexpression of candidate genes and clinical pathological parameters, including sex, age, tumor type, tumor stage, and smoking. A *P* < 0.05 was considered to be statistically significant.

## Results

### Generation of a specialized non-gapped CGH microarray representing 18 human chromosome imbalance hotspot regions

This study constructed a non-gapped BAC array covering 18 human chromosome regions including 2p22.1-p25.1, 2q34-q36.1, 3p11.2-p26.3, 4p15.1-p15.31, 4q33-q34.2, 6p12.3-p21.2, 6p22.1-p22.3, 9q21.1-q21.3, 10q21.3-25.1, 12p12.31-13.33, 12q14-q15, 13q12.3-14.2, 17p12-13.3, 17q24.2-25.3, Xp22.11, Xp22.13-22.31, Xq21.31-21.33, and Xq25-26.1 (see Additional file 2: Table S2). The chromosomal regions examined in the current array-CGH showed a high loss of heterozygosity (LOH) frequency in our previous genome-wide LOH study [13]. To construct this specialized array, genomic segments fully covering these 18 chromosome regions with 3604 BAC genomic clones without gaps were mapped. BAC clones were purified, amplified, and arrayed onto glass slides. The homogenous self-self hybridization image of reference DNA shown in the Additional file 3: Figure S1 (upper left panel) indicates the feasibility of our specialized array-CGH.

### Block-wise normalization was conducted and a Bayes regression approach was used to identify the regions with chromosomal copy number alterations

Tumor DNA from 40 Asian NSCLC patients and 20 Caucasian NSCLC patients were labeled with Cy5 (red dye) and co-hybridized with reference DNA (for Asian samples: 28 matched-normal genomic DNA pool; for Caucasian samples: ten male genomic DNA purchased from Promega) labeled with Cy3 (green dye) for array-CGH. Clinical information for the patients is shown in the Additional file 1: Table S1. A “block-wise normalization” method, which normalized the array-CGH data by the log ratio of the self-self hybridization of reference DNA according to the mean and the standard deviations of each array block, was established (lower panel, Additional file 3: Figure S1). The Bayes regression approach was used to determine the chromosomal breakpoints [17]. A region was assigned +1, 0, or −1 when its gene dosage is higher, uncertain, or lower than those of its neighbors to define the chromosome alteration regions ( Additional file 3: Figure S1), respectively. Our array-CGH data has been submitted to Gene Expression Omnibus (GEO) database of NCBI with accession number GSE21276.

### The chromosome regions show high frequency of chromosomal imbalance and significant correlation with specific cancer sub-type in Asian and Caucasian lung cancer patients

The chromosome regions with copy number alteration in 40 Asian and 20 Caucasian NSCLC patients were determined. A region with a frequency of gain or loss greater than 50% was defined as a significant chromosomal imbalance region [18,19]. In samples from Asian lung cancer patients, we have identified eight regions of chromosomal gain and nine regions of chromosomal loss (Table 1A) harboring 476 genes (UCSC Genome browser). In Caucasian lung cancer patients, eleven chromosome regions with frequent gain and nine regions with frequent loss harboring a total of 459 genes were identified (Table 1B). Interestingly, seven common chromosomal imbalance regions harboring 117 genes, included gain on 3p13-14, 6p22.1, 9q21.13, 13q14.1, and 17p13.3; and loss on 3p22.2-22.3 and 13q13.3 (Table 1A-B) were found in both Asian and Caucasian patients. In addition, when tumors were grouped into two major NSCLC tumor subtypes, adenocarcinoma (ADC) and squamous cell carcinoma (SCC), six chromosomal imbalance regions showed difference of more than 50% in alteration frequency between ADC and SCC in Asian lung cancer patients (Table 2A). In Caucasian lung cancer patients, 14 chromosome regions showed significant difference between ADC and SCC patients (Table 2B). When tumors were grouped based on the tumor stage, three chromosomal imbalance regions with difference of more than 50% in alteration frequency between early stage (stages I and II) and late stage (stages III and IV) in Asian lung cancer patients (Table 3A) were found. In Caucasian lung cancer patients, five chromosome regions showed higher alteration frequency in late stage patients than early stage patients (Table 3B). 

**Table 1:** Chromosome imbalance regions in lung cancer (alteration frequency > 50%)

**Chromosome**	**Cytoband**^**a**^	**Start site**^**b**^	**End site**^**b**^	**Size (Mb)**^**c**^	**BAC number**	**Alteration frequency (n=40)**	**Gene number**^**d**^
**A. Asian**							
3	p13-14.1*	64934270	73536726	8.60	74	Gain: 73%	23
6	p22.1*	26562439	28452189	1.89	60	Gain: 70%	31
9	q21.13*	73445866	76242707	2.80	29	Gain: 70%	8
10	q21.3	69354590	71006065	1.65	15	Gain: 55%	23
10	q24.1	95664566	99431796	3.77	37	Gain: 58%	44
12	p13.31-13.32	2363194	8268165	5.90	44	Gain: 60%	58
13	q14.1-14.2*	42340030	47097431	4.76	38	Gain: 80%	22
17	p13.3*	373082	2754327	2.38	20	Gain: 60%	37
2	p23.2-23.3	25035233	33995335	8.96	75	Loss: 63%	83
2	p24.1	21212355	23783250	2.57	18	Loss: 58%	1
2	q35	215083214	215870969	0.79	9	Loss: 58%	5
3	p21.3-22.3*	33902686	44181635	10.28	82	Loss: 65%	90
4	p15.2-15.31	22255381	24549727	2.29	24	Loss: 60%	5
6	p22.2	23982002	25939112	1.96	68	Loss: 80%	16
12	q15	67646357	69188647	1.54	11	Loss: 55%	11
13	q13.3*	35856763	39003721	3.15	27	Loss: 65%	18
X	p22.31	5701499	5872842	0.17	2	Loss: 50%	1
**Overall**							476
**B. Caucasian**							
2	p24.1-24.3	12723105	21045423	8.32	58	Gain: 55%	21
2	q25.1	11704089	12544967	0.84	7	Gain: 50%	2
3	p12.3-13*	67690502	79574641	11.88	96	Gain: 70%	22
3	p14.1-14.3*	56804094	67550231	10.75	65	Gain: 50%	26
3	p21.31-22.1	42559388	46459596	3.90	33	Gain: 60%	57
3	p25.3-26.3	1711179	9591700	7.88	72	Gain: 55%	20
6	p22.1-22.3*	20951128	28567815	7.62	132	Gain: 60%	97
9	q21.13*	75834015	76513536	0.68	6	Gain: 70%	5
13	q14.1-14.2*	40944092	47097431	6.15	47	Gain: 80%	24
17	p13.2-13.3*	481074	4001084	3.52	13	Gain: 70%	35
X	p22.11-22.2	16180300	22691476	6.51	49	Gain: 60%	26
2	p22.3	32854381	33890229	1.04	6	Loss: 50%	3
2	p24.3-25.1	31026798	31813698	0.79	6	Loss: 50%	3
3	p11.1-12.1	84572959	90247330	5.67	46	Loss: 70%	9
3	p22.2-22.3*	32980614	36795763	3.82	13	Loss: 75%	14
3	p23	31259835	32891762	1.63	13	Loss: 60%	10
6	p21.2-22.1	29049491	39602226	10.55	86	Loss: 70%	27
10	p23.1-23.2	82106537	89500949	7.39	79	Loss: 60%	36
12	p12.31	8090287	9190860	1.10	7	Loss: 50%	18
13	q13.3*	35856763	36315193	0.46	5	Loss: 80%	4
**Overall**							459

^a^ The common alteration regions in both Asian and Caucasian are marked with asterisk (*).

^b^ The information of cytoband start site and end site nucleotide sequences were from UCSC database (http://genome.ucsc.edu/, build 36 - hg18).

^c^ The sizes of cytoband were calculated from the start site to the end site nucleotide sequences.

^d^ The gene number of candidate genes was estimated by “NimbleGen: Transcription_Start_Sites.gff” annotation file (Roche, Palo Alto, CA). The annotation file indicates all transcription initiation sites for build hg18 as reported in the UCSC Genome browser.

**Table 2:** Chromosome imbalance regions with different alteration frequency in relation to tumor subtypes in lung cancer

**Chromosome**	**Cytoband**	**Start site**	**End site**	**Size (Mb)**	**BAC number**	**ADC**^**a**^** (n=20)**	**SCC**^**a**^** (n=20)**	**Difference in alteration frequency**
**A. Asian**								
2	p24.1	20863719	22199349	1.30	10	Loss :85%	Loss :30%	55%
3	p25.2-25.3	9867502	12488641	2.60	18	Loss :25%	Gain :40%	65%
6	p22.1	28165236	28452189	0.30	6	Gain :85%	Gain :25%	60%
10	q22.3	77428562	79105320	1.70	17	Gain :15%	Loss :45%	60%
10	q24.1	98845671	99207113	0.36	4	Gain :50%	Gain :70%	20%
17	p13.1	7918593	7993947	0.10	2	Loss :10%	Gain :45%	55%
17	q24.2	62769444	63340772	0.60	5	Loss :60%	0	60%
**B. Caucasian**								
2	p23.3	23804032	26257119	2.45	17	Gain :60%	0	60%
3	p11.2-12.1	85893658	87649703	1.76	14	Loss :100%	Loss :40%	60%
3	p14.2-14.3	56804094	61897044	5.09	33	Gain :20%	Gain :80%	60%
3	p22.2-24.1	29648645	38540543	8.89	25	Loss :70%	0	70%
4	p15.2-15.3	21407638	23952439	2.54	27	0	Loss :80%	80%
6	p12.3	47804985	47961964	0.16	2	0	Gain :60%	60%
6	p22.1-22.2	25985490	26318093	0.33	5	0	Gain :50%	50%
6	q21.1	73325343	73596310	0.27	2	Loss :60%	0	60%
9	q22.2-22.3	76902029	78697046	1.80	17	Loss :50%	Gain :20%	70%
10	q21.2	74105644	74697200	0.59	6	0	Gain :60%	60%
12	p13.3	8010296	9309215	1.30	11	Loss :90%	Loss :10%	80%
12	q24.2	63460935	64329625	0.87	14	Loss :10%	Loss :60%	50%
17	p22.2	15476489	15931167	0.45	4	Gain :60%	0	60%
X	q21.3	87836131	89896378	2.06	17	0	Gain :60%	60%

^a^ADC: adenocarcinoma; SCC: squamous cell carcinoma.

**Table 3:** Chromosome imbalance regions with different alteration frequency in relation to tumor stages in lung cancer

**Chromosome**	**Cytoband**	**Start site**	**End site**	**Size (Mb)**	**BAC number**	**Early stage (n=19)**	**Late Stage (n=21)**	**Difference in alteration frequency**
**A. Asian**								
6	p21.2	39548717	39877219	0.32	13	0	Loss :62%	62%
6	p22.3	21096443	22252887	1.15	39	0	Gain :66%	66%
17	p13.3	2443685	3461082	1.01	9	Gain :21%	Gain :71%	50%
**B. Caucasian**								
2	p22.2-22.3	35262506	36832851	1.57	11	0	Gain :100%	100%
2	p25.1	8929622	11112217	2.18	16	Gain :14%	Gain :100%	86%
3	p24.1-24.3	23113657	26730485	3.62	32	Loss : 7%	Loss :60%	53%
4	p15.2	25955365	26477816	0.52	4	0	Gain :60%	60%
10	q24.2-24.3	100717385	104630288	3.91	44	0	Loss :100%	100%

### The significance of altered biological processes in lung cancer

**Figure 1:**
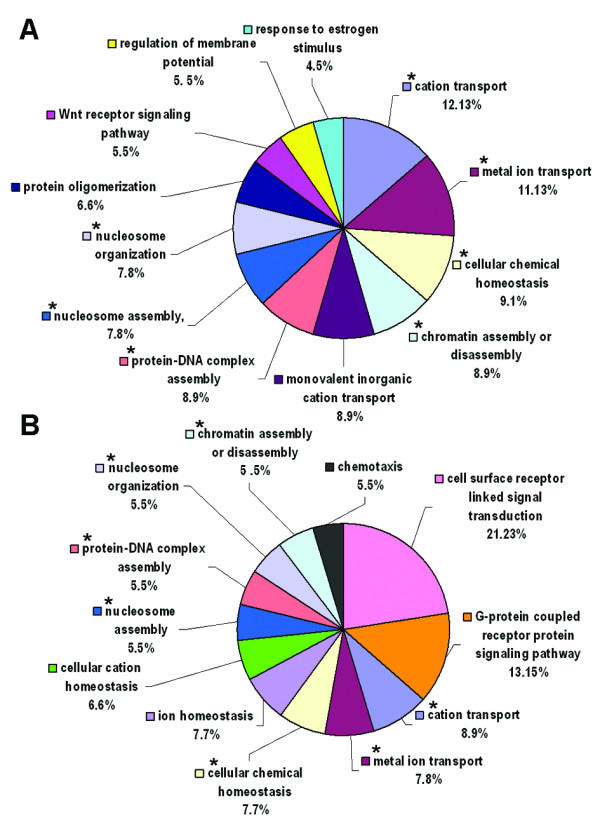
**Pie chart of significantly altered biological processes in Asian (A) and Caucasian (B) lung cancer patients.** The data were analyzed by DAVID bioinformatics resources. The common biological processes in both Asian and Caucasian marked with asterisk (*).

We then used Database for Annotation, Visualization and Integrated Discovery (DAVID) bioinformatics resources [20] to identify the difference of significantly altered biological process between Asian and Caucasian lung cancer patients. The results showed that there were seven common altered biological processes of gene ontology between Asian and Caucasian, including cation transport, metal ion transport, cellular chemical homeostasis, chromatin assembly or disassembly, protein-DNA complex assembly, nucleosome assembly, and nucleosome organization (Figure1A-B, indicated by * symbol). In addition, unique altered biological processes including monovalent inorganic cation transport, protein oligomerization, Wnt receptor signaling pathway, regulation of membrane potential, and response to estrogen stimulus were found in Asian lung cancer patients (Figure1A). Unique biological processes in Caucasian included cell surface receptor linked signal transduction, G-protein coupled receptor protein signaling pathway, ion homeostasis, cellular cation homeostasis, and chemotaxis (Figure1B). 

### A total of 134 candidate NSCLC-related genes are selected by MetaCore analysis and integration analysis with the expression array database

**Figure 2:**
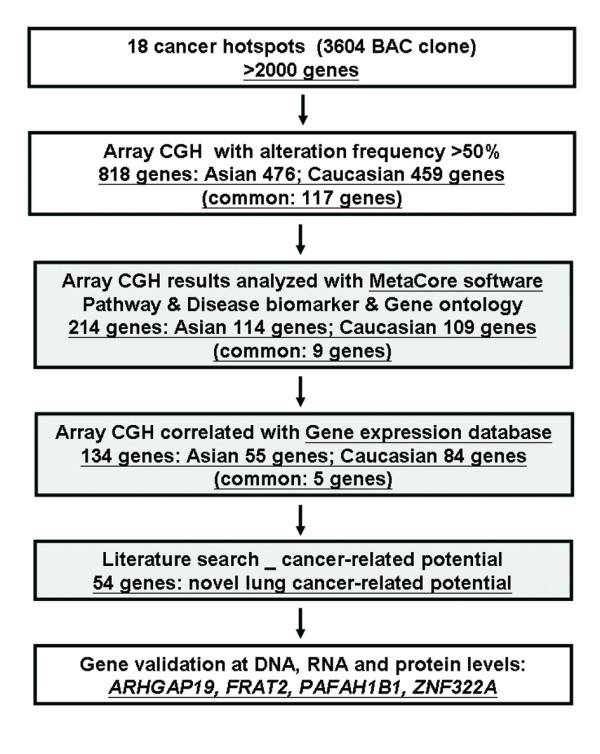
**Flowchart summarizing the strategy of candidate genes selection.** First, a total of 818 genes with more than 50% frequency of chromosome alteration were analyzed by MetaCore software. Second, the candidate genes analyzed with MetaCore were further integrated with the gene expression database. Integrating our array-CGH database with the expression array databases, 134 genes showing correlation between DNA copy number and mRNA expression patterns were found. Finally, literature search was performed to select 54 novel lung cancer candidate genes for following validation assays at the DNA, and RNA levels.

To select potential NSCLC-related genes for further studies, the following strategy of candidate gene decision was performed (Figure2). First, a total of 818 genes from Asian and Caucasian databases with chromosome alteration frequency more than 50% were further analyzed by MetaCore™ analysis software. Calculation of statistical significance throughout MetaCore for cellular pathways, disease-specific biomarker genes, and genes annotated in the gene ontology databases was based on *P* value, calculated using the hypergeometric distribution [14]. *P* value of 0.05 was used for the cutoff of candidate gene selection. Collectively, 214 genes were selected as NSCLC-related candidate genes, which are involved in cell cycle, apoptosis, cell migration, signal transduction, DNA repair and other cell growth and motility control pathways (see Additional file 5: Table S4). Second, the candidate genes analyzed with MetaCore were further integrated with the expression array for Asian lung cancer patients (GEO database with accession number GSE21933) and the gene expression database of the National Cancer Institute's Cancer Genome Anatomy Project (http://cgap.nci.nih.gov) for Caucasian lung cancer patients (see Additional file 5: Table S4). We found 134 genes showing correlation between DNA copy number and mRNA expression patterns by integrating our array-CGH database with the expression array databases (Table 4). Finally, we performed literature search and selected 54 novel lung cancer candidate genes which have not been reported to be altered in lung cancer. In the current study, we validated four genes based on their novelty and antibody availability. These four genes were *ARHGAP19* on 10q24.1 functioning in Rho activity control, *FRAT2* on 10q24.1 involved in Wnt signaling, *PAFAH1B1* on 17p13.3 functioning in motility control, and *ZNF322A* on 6p22.1 involved in MAPK pathway for following validation assays at the DNA, and RNA levels. 

**Table 4:** Candidate genes in lung cancer

**Gene name**^**a**^	**a-CGH (%)**	**Gene name**^**a**^	**a-CGH (%)**	**Gene name**^**a**^	**a-CGH (%)**		
**A. Asian**							
ACAA1	Loss: 65	FRS2	Loss: 55	RPSA	Loss: 68		
ALDH18A1	Gain: 58	HADHA	Loss: 63	RYBP*	Gain: 73		
ANKRD2	Gain: 58	HELLS	Gain: 58	SCGN	Loss: 80		
APOB	Loss: 58	HK1	Gain: 55	SERPINF1	Gain: 60		
ARHGAP19	Gain: 60	ING4	Gain: 65	SNRK	Loss: 65		
AVPI1	Gain: 58	ITGA9	Loss: 65	SPG20	Loss: 65		
AXUD1	Loss: 65	LBH	Loss: 63	SUCLG2	Gain: 73		
BLNK	Gain: 58	LRIG1	Gain: 73	TNFRSF1A	Gain: 65		
CCK	Loss: 68	LTBR	Gain: 65	TRPC4	Loss: 65		
CCNA1	Loss: 80	LYZ	Loss: 55	UBE1C*	Gain: 73		
CRK	Gain: 60	MAGI1	Gain: 73	UCN	Loss: 63		
CTDSPL	Loss: 65	MITF*	Gain: 73	VAMP1	Gain: 65		
CX3CR1	Loss: 65	MLH1	Loss: 65	YWHAE	Gain: 60		
CYP2C19	Gain: 58	MNT	Gain: 60	ZDHHC16	Gain: 58		
DLEC1	Loss: 65	MyD88	Loss: 65	ZNF322A*	Gain: 70		
FKBP4	Gain: 65	PAFAH1B1*	Gain: 60	ZNF35	Loss: 65		
FOSL2	Loss: 63	PDCD6IP	Loss: 65	ZNF384	Gain: 65		
FOXM1	Gain: 65	RAD51AP1	Gain: 65				
FRAT2	Gain: 58	RPA1	Gain: 60				
**B. Caucasian**							
**Gene name**^**a**^	**a-CGH (%)**	**Gene name**^**a**^	**a-CGH (%)**	**Gene name**^**a**^	**a-CGH (%)**	**Gene name**^**a**^	**a-CGH (%)**
ABT1	Gain: 60	HESX1	Gain: 50	PROK2	Gain: 70	VGLL3	Loss: 70
ARF4	Gain: 50	HIST1H1A	Gain: 60	PUM2	Gain: 55	WAPAL	Loss: 70
ASPA	Gain: 65	HIST1H1C	Gain: 50	RAD18	Gain: 50	XDH*	Loss: 50
CAV3	Gain: 50	HIST1H1D	Gain: 50	RBBP7	Gain: 50	YY2	Gain: 60
CCR1	Gain: 60	HIST1H1E	Gain: 50	REPS2	Gain: 50	ZBTB47	Gain: 55
CCR5	Gain: 60	HIST1H1T	Gain: 50	RhoB	Gain: 55	ZFX	Gain: 50
CD2AP	Gain: 50	HIST1H4B	Gain: 60	ROBO1	Gain: 65	ZNF165	Gain: 55
CDCP1	Gain: 60	HIST1H4F	Gain: 50	ROBO2	Gain: 70	ZNF167	Gain: 60
CGGBP1	Loss: 80	LMCD1	Gain: 50	RYBP*	Gain: 70	ZNF184	Gain: 50
CLEC3B	Gain: 60	LTF	Gain: 60	SCML1	Gain: 50	ZNF187	Gain: 55
CNTN4	Gain: 55	MBTPS2	Gain: 60	SCML2	Gain: 60	ZNF193	Gain: 55
DCLK3	Loss: 65	MDGA1	Loss: 85	SNCG	Loss: 70	ZNF197	Gain: 60
DDX1	Gain: 55	MITF*	Gain: 70	SNRK	Gain: 60	ZNF322A*	Gain: 50
DDX53	Gain: 60	MYCN	Gain: 55	SOX4	Gain: 70	ZNF35	Gain: 60
EDEM1	Gain: 50	NT5C1B	Gain: 55	SS18L2	Gain: 55	ZNF391	Gain: 50
EIF4E3	Gain: 70	NTSR2	Gain: 50	TMF1	Gain: 70	ZNF445	Gain: 60
ESD	Gain: 70	NUFIP1	Gain: 80	TNFSF11	Gain: 80	ZNF452	Gain: 50
FAM107A	Gain: 50	PAFAH1B1*	Gain: 70	TRPM6	Gain: 60	ZNF501	Gain: 60
FOXJ2	Loss: 50	PGBD1	Gain: 55	TSC22D1	Gain: 90	ZNF502	Gain: 60
FOXP1	Gain: 70	POLA1	Gain: 60	UBE1C*	Gain: 70	ZNF660	Gain: 60
GDF7	Gain: 55	PRL	Gain: 50	UBP1	Loss: 80	ZNF662	Gain: 60

^a^ The common cancer-related genes in both Asian and Caucasian are labeled with asterisk (*), and the detailed description of all genes is shown in Additional file 5: Table S4.

### A good correlation is found between array-CGH data and various validation methods including CISH, qPCR, RT-qPCR, and IHC

**Figure 3:**
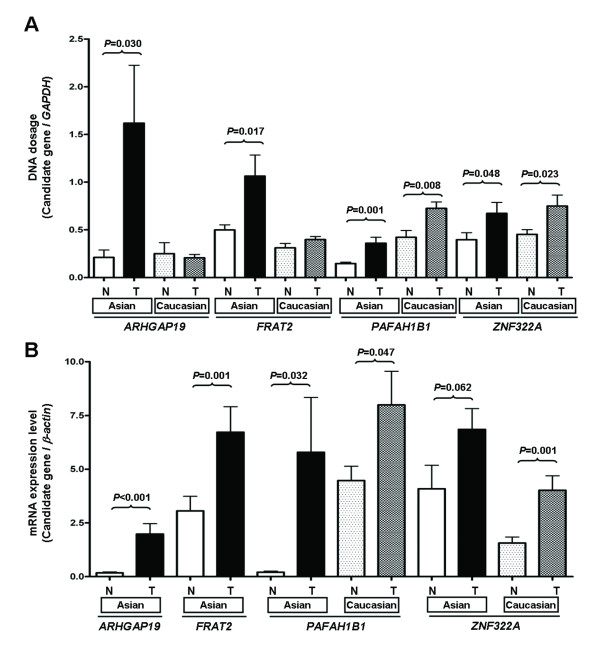
**DNA copy number validation by qPCR (A) and mRNA expression validation by RT-qPCR (B) of four candidate genes in clinical samples.** The Y-axis is the mean DNA dosage ratio (**A**) or mean mRNA expression ratio (**B**) between candidate gene and the internal control gene in all samples analyzed. The *P* values of comparison between normal (N) and tumor (T) samples for each gene is as indicated.

Four methods were employed for gene validation in 147 lung cancer patients. The clinical characteristics and the validation method conducted for each patient are shown in the Additional file 1: Table S1. qPCR for DNA copy number was performed for four candidate genes in tumors from 30 Asian and 30 Caucasian lung cancer patients, whose samples were analyzed in array-CGH. The mean gene dosage of lung cancer candidate genes *ARHGAP19*, *FRAT2, PAFAH1B1*, and *ZNF322A* in tumor tissues was significantly higher than the corresponding normal tissues in Asian lung cancer. Gene dosage of *PAFAH1B1* and *ZNF322A* was significantly higher in tumor tissues than autopsy tissues from Caucasian (Figure3A). RT-qPCR of the four genes was conducted with cDNA from tumor and corresponding normal tissues of 45 Asian lung cancer patients. Only two candidate genes, *PAFAH1B1* and *ZNF322A*, were analyzed in tumor and corresponding normal lung tissues from 24 Caucasian lung cancer patients due to the mRNA inavailability. The mean mRNA expression level of all genes analyzed in the tumor tissues was significantly higher than in the corresponding normal tissues in Asian and Caucasian patients (Figure3B). In addition, the validation data showed concordant results with array-CGH.

**Figure 4:**
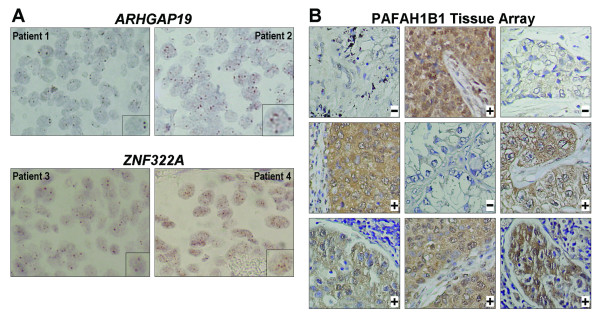
**DNA copy number and protein expression level validation in specimens from lung cancer patients.** (**A**) Representative CISH images of *ARHGAP19* and *ZNF322A* genes in paraffin-embedded lung tumor tissue. Images taken from tumor without gene copy number amplification (left) and with gene copy number amplification (right). The enlarged image of one nucleus is shown on the lower right corner. CISH images: 400X magnification. (**B**) Representative IHC images of PAFAH1B1 protein in tissue array. Patients with protein overexpression are indicated by + symbol. Patients with normal expression are indicated by – symbol. IHC images: 200X magnification.

CISH analysis of patients indicated that copy number amplification indeed occurred for *ARHGAP19* and *ZNF322A* genes in lung cancer patients (Figure4A). To determine protein expression level of the selected candidate genes in lung cancer, IHC was performed for PAFAH1B1 protein on paraffin blocks from 28 Asian patients and tissue arrays of Caucasian patients including 37 lung cancer patients (Figure4B). The frequency of PAFAH1B1 protein overexpression was 68% in Asian and 70% in Caucasian.

## Discussion

Here, we generated a non-gapped specialized CGH microarray representing 18 human chromosome imbalance hotspot regions in lung cancer. This specialized CGH array identified the chromosomal regions whose structural alteration may play critical roles in lung tumorigenesis. Our array-CGH study provided an invaluable database of chromosomal imbalance regions and candidate genes in Asian and Caucasian lung cancer patients. CISH, qPCR, RT-qPCR, and IHC were employed for gene validation of four novel genes with amplification. The putative lung cancer-related genes identified in our array-CGH database would help in further studies on the mechanism of lung tumorigenesis.

Using the UCSC Genome browser, we have annotated a total of 476 genes for Asian patients and 459 genes for Caucasian patients showing high frequency of chromosomal imbalance to be our candidate gene database (Table 1). To improve the validity for identifying potential cancer-related gene, the genes residing within the chromosomal imbalance regions were integrated with gene expression databases. Among the genes selected, the ion transport and chromatin remodeling are the two main common biological processes altered in both Asia and Caucasian populations, suggesting that the candidate genes involved in these processes may play important roles in lung tumorigenesis. For example, irregular choline transport and metabolism have been involved in growth arrest and apoptosis in human lung ADC [21]. Dysfunction of histone methylation was also shown to contribute to human carcinogenesis [22]. Interestingly, genes involved in Wnt receptor signaling pathway were unique in Asian lung cancer, whereas Caucasian lung cancer was addictive to cell surface receptor linked signal transduction and G-protein coupled receptor protein signaling pathways (Figure1A-B). Genes involved in these pathways warrant further studies.

This study found several candidate genes which are located in the common chromosome alteration regions in both Asian and Caucasian, such as *PAFAH1B1* gene on 17p13.3. In array-CGH data, chromosome region 17p13.3 harboring *PAFAH1B1* showed gain of copy number in 60% of Asian and 70% of Caucasian patients (Table 1). In addition, *PAFAH1B1* DNA copy number and mRNA expression level in tumor tissues was significantly higher than that of the matched-normal tissues in both Asian and Caucasian lung cancer patients (Figure3). The frequency of PAFAH1B1 protein overexpression was 68% in Asian and 70% in Caucasian (Figure4). Interestingly, chromosome region 17p13.3 harboring *PAFAH1B1* gene also demonstrated association with advanced tumor stage in array-CGH data (Table 3A). Overexpression of *PAFAH1B1* mRNA was found in 59.1% of early-stage and 78.3% of late-stage lung cancer patients of Asian descent ( Additional file 6: Table S5), suggesting that the alteration of *PAFAH1B1* gene may be involved in both tumor initiation and progression. More patients will be analyzed to clarify its tumorigenic role.

We also found several candidate genes showing different alteration frequency between Asian and Caucasian patients, such as *ARHGAP19* and *FRAT2*. These two closely located (within a 39.8 kb region at 10q24.1) candidate genes, showed alteration frequency of 57.5% with gain of gene copy number in Asian (Table 1A), whereas the alteration frequency was only 20% in Caucasian patients. In addition, both qPCR and RT-qPCR confirmed significant gene amplification and mRNA overexpression in tumors from Asian but not from Caucasian lung cancer patients (Figure3A-B). Interestingly, in relation to clinical pathological parameters of Asian lung cancer patients, mRNA overexpression of *ARHGAP19* and *FRAT2* was significantly associated with lung SCC ( Additional file 6: Table S5). *ARHGAP19* encodes a Rho GTPase-activating protein that stimulates the intrinsic GTP hydrolysis activity of Rho family proteins [23]. *ARHGAP* family genes are considered to be cancer-associated genes because their genetic alterations lead to carcinogenesis through the dysregulation of Rho/Rac/Cdc42-like GTPases [24]. The up-regulation of the *FRAT2* gene has been reported in human gastric cancer [25] and has been implicated in carcinogenesis through activation of the Wnt signaling pathway [26]. Our previous study showed that more than 50% of Taiwanese lung cancer patients had alterations in proteins involved in Wnt/β-catenin signaling [27]. It is important to investigate the role of ARHGAP19 and FRAT2 alterations in SCC lung tumorigenesis and their correlations with Rho and Wnt signaling pathways.

Interestingly, *ZNF322A* on 6p22.1 showed significant gene amplification and mRNA overexpression in Caucasian, and borderline significant in Asian lung cancer patients (Figure3). The DNA copy number validation for *ZNF322A* gene was lower than the array CGH result because the BAC may harbor some other genes in addition to *ZNF322A*. *ZNF322A* has been reported to be a novel human C_2_H_2_ Krüppel-like zinc-finger protein, which regulates transcriptional activation in the MAPK signaling pathways. The Krüppel zinc-finger protein is involved in the regulation of normal cell growth, differentiation, embryogenesis, and tumorigenesis [28]. Identification of the role of *ZNF322A* in lung tumorigenesis is worthy of further study.

Note that no common tumor subtype-specific regions for ADC and SCC were found in Asian and Caucasian lung cancer patients when the genomic alteration profiles were compared (Table 2A). Even in the same racial group, extreme difference of chromosome alterations could occur between ADC and SCC. These data implicated that distinct etiological mechanism in addition to tobacco and environmental exposures are involved in lung cancer of Asian from Caucasian, supporting the epidemiologic observation that lung cancer in Asian shows relatively high percentage of non-smoking female ADC. Notably, Broët *et al*. [29] identified molecular differences between NSCLCs from East-Asian and Western European patients by using single nucleotide polymorphism microarray platform, and revealed several copy number aberrations significantly associated with ethnicity (gain on 1p36, 16p13, 16p12, and 16p11 in East-Asian; loss on 19p13 in Western European patients), *EGFR* mutation (gain on 1p36, 1p35, 7p22-21, 7p15-12, 14q31-32, 16p13, and 16p12 in *EGFR* mutant tumors; loss on 21q21-22 in *EGFR* wild-type tumors), but not to *K-ras* or *p53* mutations. Correlation of the mutational spectrum of *EGFRK-rasp53*, and *ALK* genes to our array-CGH data warrants further investigations.

## Conclusions

In conclusion, our specialized array-CGH results provide an invaluable database of specific chromosome regions about the gene copy number alterations common or differential in Caucasian and Asian lung cancer patients. Functional studies using cell, animal, and clinical models will be conducted to identify their cellular functions and etiological roles in lung tumorigenesis.

## Abbreviations

array-CGH: Array-comparative genomic hybridization; qPCR: Quantitative polymerase chain reaction; ADC: Adenocarcinoma; BAC: Bacterial artificial chromosomes; NSCLC: Non-small cell lung cancer; RT-qPCR: Reverse transcriptase-qPCR; LOH: Loss of heterozygosity; DAVID: Database for Annotation, Visualization and Integrated Discovery.

## Competing interests

The authors declare that they have no competing interests.

## Authors’ contributions

YCW and FYL designed this study. FYL and JWC performed research. ISC, FUT, and SSJ analyzed array data. HSH, RK, SN, and RS provided clinical samples. YCW ,YJC, and SFH provided experimental materials. YCW and FYL wrote the manuscript. All authors read and approved the final manuscript.

## Pre-publication history

The pre-publication history for this paper can be accessed here:

http://www.biomedcentral.com/1471-2407/12/235/prepub

## Supplementary Material

Additional file 1 **Table S1.**Overall list of lung cancer patient in array-CGH, and candidate gene validations. Click here for file

Additional file 2 **Table S2.**The selected 18 chromosome regions investigated in the current array-CGH study. Click here for file

Additional file 3 **Figure S1.**Array image and schematic illustrations of the Bayes regression approach for identifying the regions with chromosomal copy number changes. Click here for file

Additional file 4 **Table S3.**Primers and PCR conditions used in the current study. Click here for file

Additional file 5 **Table S4.**List of candidate genes in Asian and Caucasian lung cancer patients. Click here for file

Additional file 6 **Table S5.**Association between mRNA expression level and lung cancer clinicopathological parameters of four candidate genes in Asian. Click here for file
