# Supplementary material for: The database of chromosome imbalance regions and genes resided in lung cancer from Asian and Caucasian identified by array-comparative genomic hybridization
Source: BMC Cancer. 2012 Jun 12;12:235. doi: 10.1186/1471-2407-12-235 (PMC3488578; doi:10.1186/1471-2407-12-235)
Supplement: Additional file 1 — Table S1.Overall list of lung cancer patient in array-CGH, and candidate gene validations. [file 1471-2407-12-235-S1.pdf]

Table S1. Overall list of lung cancer patient in array-CGH, and candidate gene validations.

Lung cancer patient [a]	Array-CGH	qPCR	CISH	RT-qPCR	IHC	Reference sample [b]	Tumor type [c]	Tumor stage
Asian_1	√	√	√	√		Matched-Normal	SCC	IA
Asian_2	√					Matched-Normal	SCC	IB
Asian_3	√	√	√	√		Matched-Normal	SCC	IB
Asian_4	√					Matched-Normal	ADC	IB
Asian_5	√	√		√		Matched-Normal	ADC	IIB
Asian_6	√	√		√		Matched-Normal	SCC	IB
Asian_7	√					Matched-Normal	ADC	IIIA
Asian_8	√					Matched-Normal	SCC	IIB
Asian_9	√					Matched-Normal	ADC	IB
Asian_10	√	√		√		Matched-Normal	ADC	IIIA
Asian_11	√	√	√	√		Matched-Normal	SCC	IIIA
Asian_12	√					Matched-Normal	SCC	IIB
Asian_13	√	√	√	√	√	Matched-Normal	SCC	IIIA
Asian_14	√	√		√	√	Matched-Normal	ADC	IIIA
Asian_15	√	√		√		Matched-Normal	ADC	IIIA
Asian_16	√	√	√	√		Matched-Normal	SCC	IIIA
Asian_17	√	√		√	√	Matched-Normal	ADC	IIIA
Asian_18	√					Matched-Normal	ADC	IIA
Asian_19	√	√		√		Matched-Normal	ADC	IB
Asian_20	√	√	√	√	√	Matched-Normal	SCC	IIIA
Asian_21	√					Matched-Normal	ADC	IV

Lung cancer patient [a]	Array-CGH	qPCR	CISH	RT-qPCR	IHC	Reference sample [b]	Tumor type [c]	Tumor stage
Asian_22	√					Matched-Normal	ADC	IIIB
Asian_23	√					Matched-Normal	ADC	IIIA
Asian_24	√		√			Matched-Normal	ADC	IIIA
Asian_25	√	√		√		Matched-Normal	SCC	IIIA
Asian_26	√	√		√		Matched-Normal	SCC	IIIA
Asian_27	√	√	√	√	√	Matched-Normal	ADC	IB
Asian_28	√			√	√	Matched-Normal	SCC	IA
Asian_29	√	√		√		Matched-Normal	ADC	IB
Asian_30	√	√		√		Matched-Normal	ADC	IIIA
Asian_31	√	√		√	√	Matched-Normal	ADC	IB
Asian_32	√					Matched-Normal	SCC	IIIA
Asian_33	√	√		√		Matched-Normal	SCC	IIIA
Asian_34	√		√	√		Matched-Normal	ADC	IIB
Asian_35	√			√		Matched-Normal	ADC	IB
Asian_36	√			√		Matched-Normal	SCC	IIIA
Asian_37	√		√	√	√	Matched-Normal	SCC	IIB
Asian_38	√					Matched-Normal	SCC	IIB
Asian_39	√					Matched-Normal	SCC	IIIB
Asian_40	√					Matched-Normal	SCC	IIIA
Asian_41				√	√	Matched-Normal	ADC	IB
Asian_42				√	√	Matched-Normal	ADC	IB
Asian_43				√	√	Matched-Normal	SCC	IIB
Asian_44				√	√	Matched-Normal	ADC	IV

Lung cancer patient [a]	Array-CGH	qPCR	CISH	RT-qPCR	IHC	Reference sample [b]	Tumor type [c]	Tumor stage
Asian_45				√	√	Matched-Normal	SCC	IIIA
Asian_46				√	√	Matched-Normal	ADC	IIIB
Asian_47				√	√	Matched-Normal	ADC	IV
Asian_48				√	√	Matched-Normal	SCC	IIIB
Asian_49				√	√	Matched-Normal	SCC	IA
Asian_50				√	√	Matched-Normal	ADC	IIIA
Asian_51				√	√	Matched-Normal	ADC	IIIA
Asian_52				√	√	Matched-Normal	ADC	IIB
Asian_53				√	√	Matched-Normal	ADC	IIB
Asian_54				√	√	Matched-Normal	ADC	IIIA
Asian_55				√	√	Matched-Normal	ADC	IIIA
Asian_56				√	√	Matched-Normal	ADC	ND
Asian_57				√	√	Matched-Normal	ADC	IB
Asian_58				√	√	Matched-Normal	ADC	IIA
Asian_59				√	√	Matched-Normal	ADC	IB
Asian_60				√	√	Matched-Normal	ADC	IIIB
Asian_61		√				Matched-Normal	ADC	IIIA
Asian_62		√				Matched-Normal	SCC	IIIB
Asian_63		√				Matched-Normal	ADC	IIB
Asian_64		√				Matched-Normal	ADC	IB
Asian_65		√				Matched-Normal	SCC	IB
Asian_66		√				Matched-Normal	ADC	IB
Asian_67		√				Matched-Normal	NSCLC	IB

Lung cancer patient [a]	Array-CGH	qPCR	CISH	RT-qPCR	IHC	Reference sample [b]	Tumor type [c]	Tumor stage
Asian_68		√				Matched-Normal	ADC	IB
Asian_69		√				Matched-Normal	ADC	IB
Asian_70		√				Matched-Normal	ADC	IB
Caucasian_1	√	√				Male human genomic DNA / autopsy samples	SCC	IIIA
Caucasian_2	√	√				Male human genomic DNA / autopsy samples	SCC	IA
Caucasian_3	√	√				Male human genomic DNA / autopsy samples	SCC	IIIA
Caucasian_4	√	√				Male human genomic DNA / autopsy samples	SCC	IB
Caucasian_5	√	√				Male human genomic DNA / autopsy samples	SCC	IB
Caucasian_6	√	√				Male human genomic DNA / autopsy samples	SCC	IB
Caucasian_7	√	√				Male human genomic DNA / autopsy samples	SCC	IA
Caucasian_8	√	√				Male human genomic DNA / autopsy samples	SCC	IB
Caucasian_9	√	√				Male human genomic DNA / autopsy samples	SCC	IA
Caucasian_10	√	√				Male human genomic DNA / autopsy samples	SCC	IIIA
Caucasian_11	√	√				Male human genomic DNA / autopsy samples	ADC	I
Caucasian_12	√	√				Male human genomic DNA / autopsy samples	ADC	III
Caucasian_13	√	√				Male human genomic DNA / autopsy samples	ADC	ND
Caucasian_14	√	√				Male human genomic DNA / autopsy samples	ADC	I
Caucasian_15	√	√				Male human genomic DNA / autopsy samples	ADC	I
Caucasian_16	√	√				Male human genomic DNA / autopsy samples	ADC	I
Caucasian_17	√	√				Male human genomic DNA / autopsy samples	ADC	III
Caucasian_18	√	√				Male human genomic DNA / autopsy samples	ADC	I
Caucasian_19	√	√				Male human genomic DNA / autopsy samples	ADC	II
Caucasian_20	√	√				Male human genomic DNA / autopsy samples	ADC	I

Lung cancer patient [a]	Array-CGH	qPCR	CISH	RT-qPCR	IHC	Reference sample [b]	Tumor type [c]	Tumor stage
Caucasian_21					√	Surrounding-Normal	LCC	III
Caucasian_22					√	Surrounding-Normal	ADC	III
Caucasian_23					√	Surrounding-Normal	LCC	III
Caucasian_24					√	Surrounding-Normal	LCC	III
Caucasian_25					√	Surrounding-Normal	ADC	IV
Caucasian_26					√	Surrounding-Normal	ADC	III
Caucasian_27					√	Surrounding-Normal	LCC	III
Caucasian_28					√	Surrounding-Normal	ADC	IIIA
Caucasian_29					√	Surrounding-Normal	SCC	III
Caucasian_30					√	Surrounding-Normal	SCLC	III
Caucasian_31					√	Surrounding-Normal	SCC	II
Caucasian_32					√	Surrounding-Normal	LCC	III
Caucasian_33					√	Surrounding-Normal	LCC	III
Caucasian_34					√	Surrounding-Normal	LCC	I
Caucasian_35					√	Surrounding-Normal	ADC	II
Caucasian_36					√	Surrounding-Normal	LCC	III
Caucasian_37					√	Surrounding-Normal	ADC	III
Caucasian_38					√	Surrounding-Normal	LCC	III
Caucasian_39					√	Surrounding-Normal	ADC	III
Caucasian_40					√	Surrounding-Normal	ADC	II
Caucasian_41					√	Surrounding-Normal	LCC	I
Caucasian_42					√	Surrounding-Normal	LCC	I
Caucasian_43					√	Surrounding-Normal	LCC	I

Lung cancer patient [a]	Array-CGH	qPCR	CISH	RT-qPCR	IHC	Reference sample [b]	Tumor type [c]	Tumor stage
Caucasian_44					√	Surrounding-Normal	LCC	I
Caucasian_45					√	Surrounding-Normal	LCC	I
Caucasian_46					√	Surrounding-Normal	LCC	I
Caucasian_47					√	Surrounding-Normal	LCC	II
Caucasian_48					√	Surrounding-Normal	LCC	ND
Caucasian_49					√	Surrounding-Normal	LCC	I
Caucasian_50					√	Surrounding-Normal	LCC	I
Caucasian_51					√	Surrounding-Normal	SCLC	II
Caucasian_52					√	Surrounding-Normal	LCC	I
Caucasian_53					√	Surrounding-Normal	LCC	I
Caucasian_54					√	Surrounding-Normal	LCC	I
Caucasian_55					√	Surrounding-Normal	SCLC	II
Caucasian_56					√	Surrounding-Normal	LCC	I
Caucasian_57					√	Surrounding-Normal	LCC	I
Caucasian_58		√				autopsy samples	SCC	ND
Caucasian_59		√				autopsy samples	SCC	ND
Caucasian_60		√				autopsy samples	SCC	ND
Caucasian_61		√				autopsy samples	SCC	ND
Caucasian_62		√				autopsy samples	SCC	ND
Caucasian_63		√				autopsy samples	SCC	ND
Caucasian_64		√				autopsy samples	ADC	IIIA
Caucasian_65		√				autopsy samples	ADC	IIIA
Caucasian_66		√				autopsy samples	ADC	IA

Lung cancer patient [a]	Array-CGH	qPCR	CISH	RT-qPCR	IHC	Reference sample [b]	Tumor type [c]	Tumor stage
Caucasian_67		√				autopsy samples	ADC	IIIA
OriGene_pair_1				√		Matched-Normal	SCC	IA
OriGene_pair_2				√		Matched-Normal	ADC	IA
OriGene_pair_3				√		Matched-Normal	LCC	IA
OriGene_pair_4				√		Matched-Normal	NSCLC	IA
OriGene_pair_5				√		Matched-Normal	LCC	IB
OriGene_pair_6				√		Matched-Normal	LCC	IB
OriGene_pair_7				√		Matched-Normal	ADC	IB
OriGene_pair_8				√		Matched-Normal	ADC	IB
OriGene_pair_9				√		Matched-Normal	SCC	IIA
OriGene_pair_10				√		Matched-Normal	SCC	IIA
OriGene_pair_11				√		Matched-Normal	NSCLC	IIB
OriGene_pair_12				√		Matched-Normal	SCC	IIB
OriGene_pair_13				√		Matched-Normal	SCC	IIB
OriGene_pair_14				√		Matched-Normal	ADC	IIB
OriGene_pair_15				√		Matched-Normal	ADC	IIB
OriGene_pair_16				√		Matched-Normal	SCC	IIB
OriGene_pair_17				√		Matched-Normal	NSCLC	IIB
OriGene_pair_18				√		Matched-Normal	SCC	IIB
OriGene_pair_19				√		Matched-Normal	ADC	IIIA
OriGene_pair_20				√		Matched-Normal	ADC	IIIA
OriGene_pair_21				√		Matched-Normal	ADC	IIIA
OriGene_pair_22				√		Matched-Normal	ADC	IIIB

Lung cancer patient [a]	Array-CGH	qPCR	CISH	RT-qPCR	IHC	Reference sample [b]	Tumor type [c]	Tumor stage
OriGene_pair_23				√		Matched-Normal	ADC	IIIB
OriGene_pair_24				√		Matched-Normal	ADC	IIIB
**N number of Asian**	**40**	**30 (20)** [d]	**10 (10)**	**45 (25)**	**28 (8)**	**N= 56 for gene validations**		
**N number of Caucasian**	**20**	**30 (20)**	**0 (0)**	**24 (0)**	**37 (0)**	**N= 91 for gene validations**		
**Overall**	**60**	**60 (40)**	**10 (10)**	**69 (25)**	**65 (8)**	**N= 147 for gene validations**		

[a] Surgically resected tumor tissue and matched-normal tissue were collected from 70 patients with primary NSCLC admitted to Taipei Veterans General Hospital, Taiwan and 67 tumor tissues from Caucasian patients obtained from the University of Chicago, USA. In addition, TissueScan LUNG CANCER Tissue qPCR Panel IV (OriGene, USA) was used for Caucasian RT-qPCR validation.

[b] Due to the unavailability of Caucasian clinical samples, the Male human genomic DNA (Promega) was used as the reference DNA for array-CGH. The DNA from 12 autopsy samples provided from the University of Chicago was used as the reference DNA for qPCR.

[c] ADC: adenocarcinoma; SCC: squamous cell carcinoma; LCC: large cell lung cancer; NSCLC: non-small cell lung cancer; SCLC: small cell lung cancer; ND: non-determined.

[d] The number of validation samples, which also used for array-CGH analysis were shown in brackets.
