# Supplementary material for: The database of chromosome imbalance regions and genes resided in lung cancer from Asian and Caucasian identified by array-comparative genomic hybridization
Source: BMC Cancer. 2012 Jun 12;12:235. doi: 10.1186/1471-2407-12-235 (PMC3488578; doi:10.1186/1471-2407-12-235)
Supplement: Additional file 2 — Table S2.The selected 18 chromosome regions investigated in the current array-CGH study. [file 1471-2407-12-235-S2.pdf]

Table S2. The selected 18 chromosome regions investigated in the current array-CGH study.

Chromosome	Cytoband	Length (Mb)	BAC number	LOH frequency[a]
2	2p22.1-25.1	30.4	259	47%
	2q34-36.1	12.2	154	64%
3	3p11.2-26.3	90.3	810	47%
4	4p15.1-15.31	11.2	128	56%
	4q33-34.2	10.4	96	51%
6	6p12.3-21.2	11.7	436	56%
	6p22.1-22.3	8.3	383	43%
9	9q21.1-21.3	8.6	85	47%
10	10q21.3-25.1	40.5	426	37%
12	12p13.1-13.33	11.0	99	45%
	12q14.1-15	9.0	74	59%
13	13q12.3-14.2	11.0	115	62%
17	17p12-13.3	13.7	118	57%
	17q24.2-25.3	17.0	150	47%
X	Xp22.11	3.1	23	--
	Xp22.31-22.13	13.2	94	--
	Xq21.31-21.33	12.1	84	--
	Xq25-26.1	9.4	70	--
Overall	18	323.1	3604	>45%

[a] LOH frequency in some selected chromosome region is less than 45% because these regions are important chromosome regions associated with clinicopathological parameters in our previous LOH study [1].

References

1. Tseng RC, Chang JW, Hsien FJ, Chang YH, Hsiao CF, Chen JT, Chen CY, Jou YS, Wang YC: **Genomewide loss of heterozygosity and its clinical associations in non small cell lung cancer**. *Int J Cancer* 2005, **117**:241-247.
