# Supplementary material for: The database of chromosome imbalance regions and genes resided in lung cancer from Asian and Caucasian identified by array-comparative genomic hybridization
Source: BMC Cancer. 2012 Jun 12;12:235. doi: 10.1186/1471-2407-12-235 (PMC3488578; doi:10.1186/1471-2407-12-235)
Supplement: Additional file 3 — Figure S1.Array image and schematic illustrations of the Bayes regression approach for identifying the regions with chromosomal copy number changes. [file 1471-2407-12-235-S3.pdf]

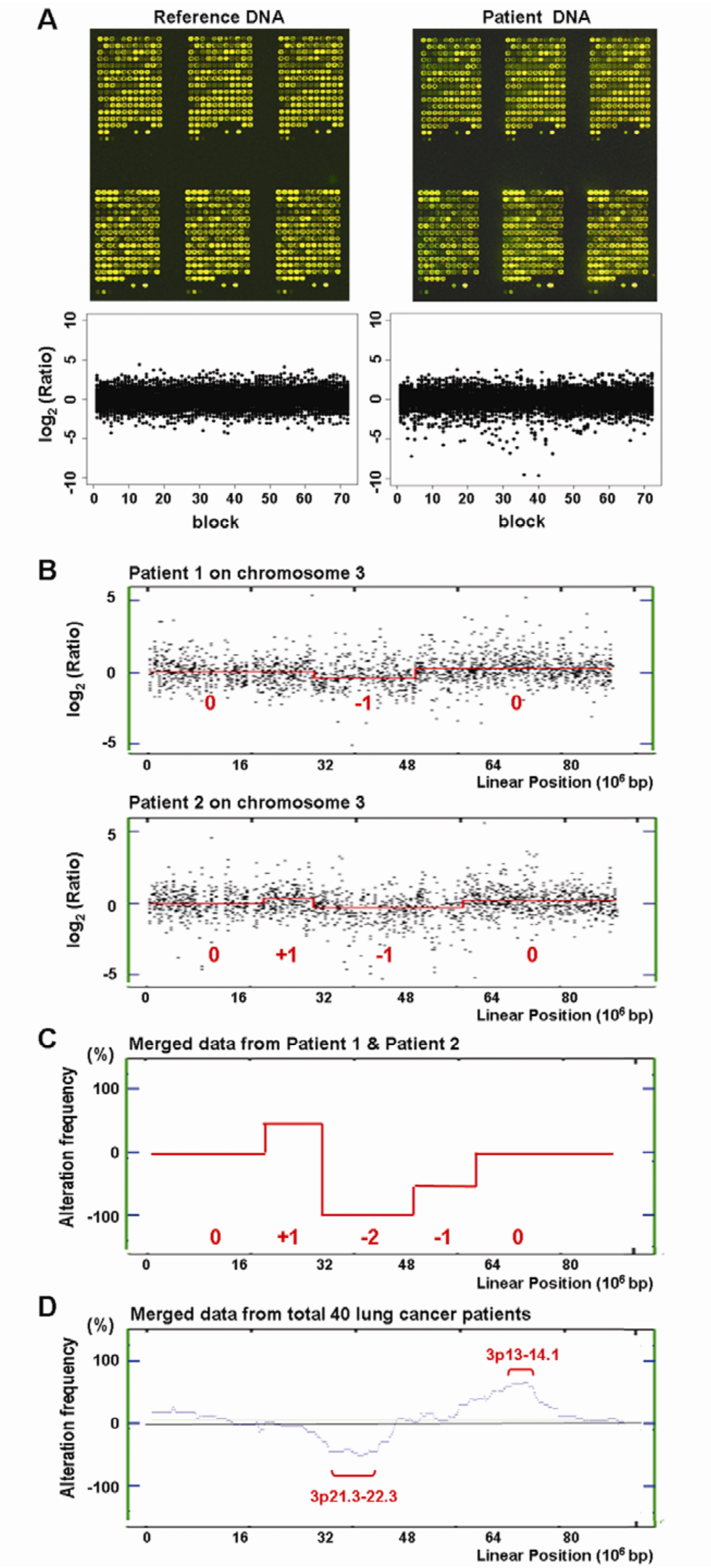

A
Reference DNA
Patient DNA
log2 (Ratio)
block
B
Patient 1 on chromosome 3
0
-1
0
Linear Position (10^6 bp)
Patient 2 on chromosome 3
0
+1
-1
0
Linear Position (10^6 bp)
C
Merged data from Patient 1 & Patient 2
(%)
Alteration frequency
0
+1
-2
-1
0
Linear Position (10^6 bp)
D
Merged data from total 40 lung cancer patients
(%)
Alteration frequency
3p13-14.1
3p21.3-22.3
Linear Position (10^6 bp)

Figure S1. Block-wise normalization was conducted and the Bayes regression approach was used to identify the regions with chromosomal copy number changes. **(A)** Array images of self-hybridization of reference DNA (left) and co-hybridization of patient DNA and reference DNA (right) are shown. The log_2 ratio of these two arrays after block-wise normalization is shown in the lower panels. The log_2 ratio of array of lung cancer patient was more deviated from 0 than that of reference sample. **(B)** The schematic illustrations show the log_2 ratio of all BAC probes on chromosome 3 in two patients. The values of +1, 0, or -1 were assigned to a region where gene dosage is higher, uncertain, or lower than those of the neighboring regions, respectively. **(C)** Merged data of alteration frequency, in which the gain chromosome region with 50% frequency (+1) at the position 20~32 10^6 bp and the loss chromosome region with 100% frequency (-2) at the position 32~48 10^6 bp on chromosome 3, for patient 1 and patient 2 is shown. **(D)** Data from a total of 40 lung cancer patients were merged, and the results show two alteration regions with gain at 3p13-14.1 and loss at 3p21.3-22.3.
