# Supplementary material for: The database of chromosome imbalance regions and genes resided in lung cancer from Asian and Caucasian identified by array-comparative genomic hybridization
Source: BMC Cancer. 2012 Jun 12;12:235. doi: 10.1186/1471-2407-12-235 (PMC3488578; doi:10.1186/1471-2407-12-235)
Supplement: Additional file 4 — Table S3.Primers and PCR conditions used in the current study. [file 1471-2407-12-235-S4.pdf]

Table S3. Primers and PCR conditions used in the current study.

Gene	Primers	5’→3’ sequences	Application	PCR size	Tm
ZNF322A	Forward<br>Reverse	TCA GTG AGG GGA AAT CTT GG<br>TTC TTC TGC CTT GAG GTG CT	qPCR	265bp	60°C
FRAT2	Forward<br>Reverse	ATG CAT GAG AGA CCC TGG AC<br>TCA GGA CCA CCA CAA TTT CA	qPCR	329bp	58 ^{o}C
PAFAH1B1	Forward<br>Reverse	TCT GGC TCA GTG TGA TGG AG<br>TGA TCT ACG CTG CCA GTG AC	qPCR	320bp	58 ^{o}C
ARHGAP19	Forward<br>Reverse	GGC TCA TCG ATT TAC CTG GA<br>GAT GCC CCG ATG ACT ACC TA	qPCR	383bp	58 ^{o}C
GAPDH	Forward<br>Reverse	AAA CTT AGC AAC CGG CTT TTA GC<br>GGG CGA AGA GCG GGA	qPCR	401bp	58 ^{o}C
ZNF322A	Forward<br>Reverse	GAA GCC CAG ATT CGA GTG TC<br>GTT TTC CGC TGG TCT TCT TG	RT-qPCR	389bp	58 ^{o}C
FRAT2	Forward<br>Reverse	ATG CAT GAG AGA CC CTGG AC<br>TCA GGA CCA CCA CAA TTT CA	RT-qPCR	329bp	58 ^{o}C
PAFAH1B1	Forward<br>Reverse	ATG GGT CGT AGC AAC AAA GG<br>TCT TCA TGC ATC GCT TGT TC	RT-qPCR	344bp	58 ^{o}C
ARHGAP19	Forward<br>Reverse	CAC CAT ACG GAA GAG GCA CT<br>GTC ATC GTG ACA GCT GGA GA	RT-qPCR	329bp	58 ^{o}C
β-actin	Forward<br>Reverse	GGC GGC ACC ACC ATG TAC CCT<br>AGG GGC CGG ACT CGT CAT ACT	RT-qPCR	314bp	58 ^{o}C
