# Supplementary material for: The database of chromosome imbalance regions and genes resided in lung cancer from Asian and Caucasian identified by array-comparative genomic hybridization
Source: BMC Cancer. 2012 Jun 12;12:235. doi: 10.1186/1471-2407-12-235 (PMC3488578; doi:10.1186/1471-2407-12-235)
Supplement: Additional file 5 — Table S4.List of candidate genes in Asian and Caucasian lung cancer patients. [file 1471-2407-12-235-S5.pdf]

Table S4. List of candidate genes in Asian and Caucasian lung cancer patients[a].

Code	Asian gene name	Accession	Gene full name	Cytoband	Array CGH alteration freq.(%)	Correlation with expression array	Cellular pathway / Disease biomarker / Gene ontology analysis from MetaCore
1	ABR	NM_001092	active BCR-related gene	17p13.3	Gain: 60	No	Neoplasms, glandular and epithelial
2	ACAA1	NM_001607	acetyl-Coenzyme A acyltransferase 1 (peroxisomal 3-oxoacyl-Coenzyme A thiolase)	3p22.2-3	Loss: 65	Yes	Neoplasms, glandular and epithelial, Adenocarcinoma, Carcinoma
3	ACVR2B	NM_001106	activin A receptor, type IIB	3p22.2-3	Loss: 65	No	Regulation of kinase activity
4	ALDH18A1	NM_001017423	aldehyde dehydrogenase 18 family, member A1	10q24.1	Gain: 57.5	Yes	Carbohydrates metabolism/ Propionate metabolism
5	ALDH1A1	NM_000689	aldehyde dehydrogenase 1 family, member A1	9q21.13	Gain: 70	No	Aminoacid metabolism/ Tryptophan metabolism, Vitamin and cofactor metabolism/ Retinol metabolism
6	ALK	NM_004304	anaplastic lymphoma kinase (Ki-1)	2p22.2-23.3	Loss: 62.5	No	Respiratory tract neoplasm, thoracic neoplasms, chromosome abberation, neoplasms, glandular and epithelial, adenocarcinoma, lung neoplasms, carcinoma
7	ANKRD2	NM_020349	ankyrin repeat domain 2 (stretch responsive muscle)	10q24.1	Gain: 57.5	Yes	Adenoma, neoplasms, glandular and epithelial, Adenocarcinoma, Carcinoma

8	ANXA1	NM_000700	annexin A1	9q21.13	Gain: 70	No	Respiratory tract neoplasm, thoracic neoplasms, neoplasms, glandular and epithelial, adenocarcinoma, lung neoplasms, carcinoma, squamous cell carcinoma, squamous cell / regulation of apoptosis, negative regulation of apoptosis, regulation of programmed cell death, negative regulation of programmed cell death, negative regulation of cell death, anti-apoptosis
9	APOB	NM_000384	apolipoprotein B (including Ag(x) antigen)	2p24.1	Loss: 57.5	Yes	Vitamin and cofactor metabolism/ Retinol metabolism
10	ARHGAP19	NM_032900	Rho GTPase activating protein 19	10q24.1	Gain: 60	Yes	Signal transduction
11	ARL6IP5	NM_006407	ADP-ribosylation-like factor 6 interacting protein 5	3p13	Gain: 72.5	No	Neoplasms, glandular and epithelial, Carcinoma, Carcinoma, Squamous Cell
12	AVPI1	NM_021732.2	arginine vasopressin-induced 1	10q24.1	Gain: 57.5	Yes	Regulation of MAP kinase activity, activation of MAPK activity, regulation of kinase activity
13	AXUD1	NM_033027	AXIN1 up-regulated 1	3p22.2-3	Loss: 65	Yes	Respiratory tract neoplasm, thoracic neoplasms, neoplasms, glandular and epithelial, adenocarcinoma, lung neoplasms, carcinoma / cell death, apoptosis
14	BARD1	NM_000465	BRCA1 associated RING domain 1	2q35	Loss: 57.5	No	DNA-damage/ Role of Brca1 and Brca2 in DNA repair / neoplasms, glandular and epithelial, Adenocarcinoma, Carcinoma / regulation of apoptosis, negative regulation of apoptosis, regulation of programmed cell death, negative regulation of programmed cell death, negative regulation of cell death

15	Birc6	NM_016252	baculoviral IAP repeat-containing 6 (apollon)	2p22.2-23.3	Loss: 62.5	No	Regulation of apoptosis, negative regulation of apoptosis, regulation of programmed cell death, cell death, negative regulation of programmed cell death, negative regulation of cell death, anti-apoptosis, apoptosis
16	BLNK	NM_013314	B-cell linker	10q24.1	Gain: 57.5	Yes	Chromosome aberration
17	BRE	NM_199193	brain and reproductive organ-expressed (TNFRSF1A modulator)	2p22.2-23.3	Loss: 62.5	No	Neoplasms, glandular and epithelial, adenocarcinoma, carcinoma / DNA damage checkpoint, cell death, DNA integrity checkpoint, double-strand break repair, regulation of cell cycle, apoptosis
18	CCK	NM_000729	cholecystokinin	3p22.1	Loss: 67.5	Yes	Neoplasms, glandular and epithelial, Tobacco Use Disorder / regulation of apoptosis, regulation of programmed cell death, cell death, regulation of kinase activity, apoptosis
19	CCNA1*	NM_003914	cyclin A1	13q13.3	Loss: 80	Yes	Cell cycle/ regulation of G2/S transition, cell cycle/ chromosome condensation in prometaphase / adenocarcinoma
20	CCND2	NM_001759	cyclin D2	12p13.3	Gain: 65	No	Respiratory tract neoplasm, thoracic neoplasms, neoplasms, glandular and epithelial, adenocarcinoma, lung neoplasms, carcinoma, carcinoma, squamous cell / regulation of kinase activity, regulation of cell cycle, positive regulation of epithelial cell proliferation

21	CD27	NM_001242	CD27 molecule	12p13.3	Gain: 65	ND	Regulation of apoptosis, negative regulation of apoptosis, regulation of programmed cell death, cell death, negative regulation of programmed cell death, negative regulation of cell death, anti-apoptosis, apoptosis
22	CD9	NM_001769	CD9 antigen (p24)	12p13.3	Gain: 65	No	Respiratory tract neoplasm, thoracic neoplasms, neoplasms, glandular and epithelial, adenocarcinoma, lung neoplasms, carcinoma, carcinoma, squamous cell
23	CDCP1	NM_022842	CUB domain-containing protein 1	3p22.1-21.33	Loss: 65	No	Respiratory tract neoplasm, thoracic neoplasms, neoplasms, glandular and epithelial, adenocarcinoma, lung neoplasms, carcinoma
24	CENPA	NM_001809	centromere protein A	2p22.2-23.3	Loss: 62.5	No	Chromosome aberration
25	CLASP2*	NM_015097	cytoplasmic linker associated protein 2	3p22.2-3	Loss: 65	No	Chromosome aberration / regulation of microtubule cytoskeleton organization, cell migration
26	CPB2	NM_001872	carboxypeptidase B2 (plasma, carboxypeptidase U)	13q14.1-2	Gain: 80	No	Respiratory tract neoplasm, thoracic neoplasms, lung neoplasms
27	CRK	NM_005206	v-crk sarcoma virus CT10 oncogene homolog (avian)	17p13.3	Gain: 60	Yes	Respiratory tract neoplasm, thoracic neoplasms, lung neoplasms / regulation of MAP kinase activity, activation of MAPK activity, regulation of kinase activity
28	CTDSPL	NM_001008392	CTD (carboxy-terminal domain, RNA polymerase II, polypeptide A) small phosphatase-like	3p22.2-3	Loss: 65	Yes	Respiratory tract neoplasm, thoracic neoplasms, lung neoplasms, carcinoma

29	CTNNB1	NM_001098209	catenin (cadherin-associated protein), beta 1, 88kDa	3p22.1	Loss: 67.5	No	Respiratory tract neoplasm, thoracic neoplasms, chromosome aberration, adenoma, neoplasms, glandular and epithelial, adenocarcinoma, lung neoplasms, carcinoma, genomic instability, carcinoma, squamous cell / cell death. positive regulation of epithelial cell proliferation, regulation of epithelial cell differentiation, apoptosis
30	CX3CR1	NM_001337	chemokine (C-X3-C motif) receptor 1	3p22.2-3	Loss: 65	Yes	Neoplasms, glandular and epithelial, adenocarcinoma, carcinoma / regulation of apoptosis, negative regulation of apoptosis, regulation of programmed cell death, negative regulation of programmed cell death, negative regulation of cell death, negative regulation of angiogenesis
31	CYP2C18	NM_000772	cytochrome P450, family 2, subfamily C, polypeptide 18	10q24.1	Gain: 57.5	No	Vitamin and cofactor metabolism/ retinol metabolism
32	CYP2C19	NM_000769	cytochrome P450, family 2, subfamily C, polypeptide 19	10q24.1	Gain: 57.5	Yes	steroid metabolism/ estrogen biosynthesis, vitamin and cofactor metabolism/ retinol metabolism / respiratory tract neoplasm, thoracic neoplasms, neoplasms, glandular and epithelial, adenocarcinoma, lung neoplasms, carcinoma
33	CYP2C8	NM_000770	cytochrome P450, family 2, subfamily C, polypeptide 8	10q24.1	Gain: 57.5	No	Steroid metabolism/ estrogen biosynthesis, vitamin and cofactor metabolism/ retinol metabolism

34	CYP2C9	NM_000771	cytochrome P450, family 2, subfamily C, polypeptide 9	10q24.1	Gain: 57.5	No	Steroid metabolism/ estrogen biosynthesis, vitamin and cofactor metabolism/ retinol metabolism / respiratory tract neoplasm, thoracic neoplasms, neoplasms, glandular and epithelial, lung neoplasms, carcinoma
35	DLEC1	NM_007337	deleted in lung and esophageal cancer 1	3p22.2-3	Loss: 65	Yes	Respiratory tract neoplasm, thoracic neoplasms, neoplasms, glandular and epithelial, adenocarcinoma, lung neoplasms, carcinoma / regulation of cell cycle
36	DNAJC15	NM_013238	DnaJ (Hsp40) homolog, subfamily C, member 15	13q14.1-2	Gain: 80	ND	Neoplasms, glandular and epithelial
37	DNMT3A	NM_022552	DNA (cytosine-5-)-methyltransferase 3 alpha	2p22.2-23.3	Loss: 62.5	No	Respiratory tract neoplasm, thoracic neoplasms, neoplasms, glandular and epithelial, lung neoplasms, carcinoma, carcinoma, squamous cell
38	DPH1	NM_001383	DPH1 homolog (S. cerevisiae)	17p13.3	Gain: 60	ND	regulation of cell cycle
39	DPYSL5	NM_020134	dihydropyrimidinase-like 5	2p22.2-23.3	Loss: 62.5	No	Respiratory tract neoplasm, thoracic neoplasms, neoplasms, glandular and epithelial, adenocarcinoma, lung neoplasms, carcinoma
40	FAM48A	NM_017569	family with sequence similarity 48, member A	13q13.3	Loss: 65	No	Neoplasms, glandular and epithelial, Carcinoma
41	FGF6	NM_020996	fibroblast growth factor 6	12p13.3	Gain: 65	No	Neoplasms, glandular and epithelial
42	FKBP4	NM_002014	FK506 binding protein 4, 59kDa	12p13.3	Gain: 65	Yes	Neoplasms, glandular and epithelial, adenocarcinoma, carcinoma
43	FOSL2	NM_005253	FOS-like antigen 2	2p22.2-23.3	Loss: 62.5	Yes	Cell death

44	FOXM1	NM_021953	forkhead box M1	12p13.3	Gain: 65	Yes	Neoplasms, glandular and epithelial, adenocarcinoma, carcinoma
45	FOXP1*	NM_032682	forkhead box P1	3p13	Gain: 72.5	No	Chromosome aberration, neoplasms, glandular and epithelial, adenocarcinoma, carcinoma / positive regulation of epithelial cell proliferation
46	FRAT1	NM_005479	frequently rearranged in advanced T-cell lymphomas	10q24.1	Gain: 57.5	No	Neoplasms, glandular and epithelial, adenocarcinoma, carcinoma
47	FRAT2	NM_012083	frequently rearranged in advanced T-cell lymphomas 2	10q24.1	Gain: 57.5	Yes	Cell proliferation
48	FRS2	NM_001042555	fibroblast growth factor receptor substrate 2	12q15	Loss: 55	Yes	Regulation of MAP kinase activity, activation of MAPK activity, regulation of kinase activity
49	GBA3	NM_020973	glucosidase, beta, acid 3 (cytosolic)	4p15.31-2	Loss: 60	No	Neoplasms, glandular and epithelial, Adenocarcinoma, Carcinoma
50	GMNN	NM_015895	geminin, DNA replication inhibitor	6p22.2	Loss: 80	No	Neoplasms, glandular and epithelial, adenocarcinoma, carcinoma / regulation of cell cycle
51	HADHA	NM_000182	hydroxyacyl-Coenzyme A dehydrogenase/3-ketoacyl-Coenzyme A thiolase/enoyl-Coenzyme A hydratase (trifunctional protein), alpha subunit	2p22.2-23.3	Loss: 62.5	Yes	Amino acid metabolism/ tryptophan metabolism, amino acid metabolism/ tyrosine metabolism, carbohydrates metabolism/ propionate metabolism, regulation of lipid metabolism/ regulation of fatty acid synthesis: NLTP and EHHADH

52	HADHB	NM_000183	hydroxyacyl-Coenzyme A dehydrogenase/3-ketoacyl-Coenzyme A thiolase/enoyl-Coenzyme A hydratase (trifunctional protein), beta subunit	2p22.2-23.3	Loss: 62.6	No	Amino acid metabolism/ tryptophan metabolism, amino acid metabolism/ tyrosine metabolism, carbohydrates metabolism/ propionate metabolism, regulation of lipid metabolism/ regulation of fatty acid synthesis: NLTP and EHHADH, carbohydrates metabolism/ propionate metabolism, regulation of lipid metabolism/ regulation of fatty acid synthesis: NLTP and EHHADH
53	HELLS	NM_018063	helicase, lymphoid-specific	10q24.1	Gain: 57.5	Yes	Regulation of apoptosis, negative regulation of apoptosis, regulation of programmed cell death, negative regulation of programmed cell death, negative regulation of cell death, anti-apoptosis
54	HIC1	NM_006497	hypermethylated in cancer 1	17p13.3	Gain: 60	No	Neoplasms, glandular and epithelial / regulation of cell cycle
55	HIST1H1B	NM_005322	histone 1, H1b	6p22.1	Gain: 70	No	Cell cycle/ chromosome condensation in prometaphase
56	HK1	NM_000188	hexokinase 1	10q21.3	Gain: 55	Yes	Regulation of apoptosis, negative regulation of apoptosis, regulation of programmed cell death, negative regulation of programmed cell death, negative regulation of cell death
57	HTR2A	NM_000621	5-hydroxytryptamine (serotonin) receptor 2A	13q14.1-2	Gain: 80	No	Neoplasms, glandular and epithelial, Adenocarcinoma, Carcinoma, Tobacco Use Disorder / regulation of MAP kinase activity, cell death, regulation of kinase activity
58	ING4	NM_016162	inhibitor of growth family, member 4	12p13.3	Gain: 65	Yes	Respiratory tract neoplasm, thoracic neoplasms, neoplasms, glandular and epithelial, adenocarcinoma,

							lung neoplasms, carcinoma / regulation of apoptosis, regulation of programmed cell death, cell death, regulation of cell cycle, apoptosis
59	ITGA9	NM_002207	integrin, alpha 9	3p22.2-3	Loss: 65	Yes	Respiratory tract neoplasm, thoracic neoplasms, neoplasms, glandular and epithelial lung neoplasms
60	KCNA5	NM_002234	potassium voltage-gated channel, shaker-related subfamily, member 5	12p13.3	Gain: 65	No	Neoplasms, glandular and epithelial
61	KHK	NM_000221	ketohexokinase (fructokinase)	2p22.2-23.3	Loss: 62.5	No	Neoplasms, glandular and epithelial, adenocarcinoma, carcinoma
62	LBH	NM_030915	likely ortholog of mouse limb-bud and heart gene	2p22.2-23.3	Loss: 62.5	Yes	Chromosome aberration
63	LRIG1	NM_015541	leucine-rich repeats and immunoglobulin-like domains 1	3p13	Gain: 72.5	Yes	Neoplasms, glandular and epithelial, adenocarcinoma, carcinoma
64	LTBR	NM_002342	lymphotoxin beta receptor (TNFR superfamily, member 3)	12p13.3	Gain: 65	Yes	Cell death, positive regulation of I-kappaB kinase/NF-kappaB cascade, apoptosis
65	LYZ	NM_000239	lysozyme (renal amyloidosis)	12q15	Loss: 55	Yes	Cell death
66	MAGI1	NM_001033057	membrane associated guanylate kinase, WW and PDZ domain containing 1	3p13	Gain: 72.5	Yes	Respiratory tract neoplasm, thoracic neoplasms, lung neoplasms
67	MAPRE3	NM_012326	microtubule-associated protein, RP/EB family, member 3	2p22.2-23.3	Loss: 62.5	No	Adenoma, neoplasms, glandular and epithelial
68	MITF*	NM_000248	microphthalmia-associated transcription factor	3p13	Gain: 72.5	Yes	Neoplasms, glandular and epithelial, carcinoma / regulation of cell differentiation, regulation of cell proliferation, regulation of gene expression

69	MLH1	NM_000249	mutL homolog 1, colon cancer, nonpolyposis type 2 (E. coli)	3p22.2-3	Loss: 65	Yes	DNA-damage/ role of Brca1 and Brca3 in DNA repair / respiratory tract neoplasm, thoracic neoplasms, chromosome abberation, neoplasms, glandular and epithelial, Adenocarcinoma, lung neoplasms, carcinoma, genomic instability, carcinoma, squamous cell / regulation of apoptosis, regulation of programmed cell death, double-strand break repair, regulation of cell cycle
70	MNT	NM_020310	MAX binding protein	17p13.3	Gain: 60	Yes	Regulation of apoptosis, regulation of programmed cell death, cell aging, regulation of cell cycle
71	MyD88	NM_002468	myeloid differentiation primary response gene (88)	3p22.2-3	Loss: 65	Yes	Positive regulation of I-kappaB kinase/NF-kappaB cascade
72	MYO1C	NM_001080950	myosin IC	17p13.3	Gain: 60	No	Neoplasms, glandular and epithelial, adenocarcinoma, carcinoma
73	NANOG	NM_024865	Nanog homeobox	12p13.31	Gain: 57.5	No	Neoplasms, glandular and epithelial, carcinoma, squamous cell / regulation of cell cycle
74	NCAPD2	NM_014865	non-SMC condensin I complex, subunit D2	12p13.3	Gain: 65	ND	Cell cycle/ chromosome condensation in prometaphase
75	NLRC4	NM_021209	NLR family, CARD domain containing 4	2p22.2-23.3	Loss: 62.5	ND	Regulation of apoptosis, regulation of programmed cell death, cell death, apoptosis
76	PAFAH1B1*	NM_015345	platelet-activating factor acetylhydrolase, isoform Ib, alpha subunit 45kDa	17p13.3	Gain: 60	Yes	Cytoskeleton organization
77	PDCD6IP	NM_013374	programmed cell death 6 interacting protein	3p22.2-3	Loss: 65	Yes	Regulation of apoptosis, regulation of programmed cell death, cell death, apoptosis

78	POMC	NM_000939	proopiomelanocortin (adrenocorticotropin/ beta-lipotropin/ alpha-melanocyte stimulating hormone/ beta-melanocyte stimulating hormone/ beta-endorphin)	2p22.2-23.3	Loss: 62.5	No	Adenoma, neoplasms, glandular and epithelial
79	PPM1G	NM_002707	protein phosphatase 1G (formerly 2C), magnesium-dependent, gamma isoform	2p22.2-23.3	Loss: 62.5	No	Neoplasms, glandular and epithelial, Adenocarcinoma, Carcinoma / regulation of MAP kinase activity, activation of MAPK activity, DNA damage checkpoint, cell death, regulation of kinase activity, DNA integrity checkpoint, regulation of cell cycle, positive regulation of I-kappaB kinase/NF-kappaB cascade, apoptosis
80	PREB	NM_013388	prolactin regulatory element binding	2p22.2-23.3	Loss: 62.5	No	Lung diseases/cystic fibrosis/ Delta508-CFTR traffic / ER-to-Golgi in CF / chromosome aberration
81	RAD51AP1	NM_006479	RAD51 associated protein 1	12p13.3	Gain: 65	Yes	Neoplasms, glandular and epithelial, adenocarcinoma, carcinoma / double-strand break repair via homologous recombination, double-strand break repair
82	RPA1	NM_002945	replication protein A1, 70kDa	17p13.3	Gain: 60	Yes	Respiratory tract neoplasm, thoracic neoplasms, lung neoplasms, genomic instability / double-strand break repair via homologous recombination, double-strand break repair
83	RPL14	NM_003973	ribosomal protein L14	3p22.1	Loss: 67.5	No	Lung diseases/cystic fibrosis/ CFTR translational fidelity

84	RPSA	NM_002295	ribosomal protein SA	3p22.1	Loss: 67.5	Yes	Lung diseases/cystic fibrosis/ CFTR translational fidelity / respiratory tract neoplasm, thoracic neoplasms, neoplasms, glandular and epithelial, lung neoplasms, carcinoma
85	RYBP*	NM_012234	RING1 and YY1 binding protein	3p13	Gain: 72.5	Yes	Cell death, apoptosis
86	SCGN	NM_006998	secretagogin, EF-hand calcium binding protein	6p22.2	Loss: 80	Yes	Adenoma, neoplasms, glandular and epithelial
87	SERPINF1	NM_002615	serine (or cysteine) proteinase inhibitor, clade F (alpha-2 antiplasmin, pigment epithelium derived factor), member 1	17p13.3	Gain: 60	Yes	Neoplasms, glandular and epithelial, adenocarcinoma, carcinoma, carcinoma, squamous cell / negative regulation of angiogenesis
88	SERPINF2	NM_000934	serine (or cysteine) proteinase inhibitor, clade F (alpha-2 antiplasmin, pigment epithelium derived factor), member 2	17p13.3	Gain: 60	No	Neoplasms, glandular and epithelial, adenocarcinoma, carcinoma, carcinoma, squamous cell
89	SFRP5	NM_003015	secreted frizzled-related protein 5	10q24.1	Gain: 57.5	No	Neoplasms, glandular and epithelial, carcinoma, carcinoma, squamous cell / cell death, apoptosis
90	SNRK	NM_001100594	SNF related kinase	3p22.1-21.33	Loss: 65	Yes	Neoplasms, glandular and epithelial, adenocarcinoma, carcinoma / regulation of apoptosis, regulation of programmed cell death
91	SPAST	NM_014946	spastin	2p22.2-23.3	Loss: 62.5	ND	Cell death
92	SPDYA	NM_182756	speedy homolog A (Xenopus laevis)	2p22.2-23.3	Loss: 62.5	ND	Regulation of kinase activity, regulation of cell cycle

93	SPG20	NM_015087	spastic paraplegia 20, spartin (Troyer syndrome)	13q13.3	Loss: 65	Yes	Cell death
94	SRGN	NM_002727	serglycin	10q21.3	Gain: 55	ND	Cell death, apoptosis
95	SS18L2	NM_016305	synovial sarcoma translocation gene on chromosome 18-like 2	3p22.1	Loss: 67.5	No	Chromosome aberration, neoplasms, glandular and epithelial, adenocarcinoma, carcinoma
96	SUCLG2	NM_003848	succinate-CoA ligase, GDP-forming, beta subunit	3p13	Gain: 72.5	Yes	Carbohydrates metabolism/ propionate metabolism
97	TACR2	NM_001057	tachykinin receptor 2	10q21.3	Gain: 55	No	Neoplasms, glandular and epithelial
98	TNFRSF1A	NM_001065	tumor necrosis factor receptor superfamily, member 1A	12p13.3	Gain: 65	Yes	Neoplasms, glandular and epithelial, adenocarcinoma, carcinoma, carcinoma, squamous cell / cell death, positive regulation of I-kappaB kinase/NF-kappaB cascade, apoptosis
99	TPT1	NM_003295	tumor protein, translationally-controlled 1	13q14.1-2	Gain: 80	No	Neoplasms, glandular and epithelial, carcinoma / regulation of apoptosis, negative regulation of apoptosis, regulation of programmed cell death, negative regulation of programmed cell death, negative regulation of cell death, anti-apoptosis
100	TRAK1	NM_001042646	trafficking protein, kinesin binding 1	3p22.1	Loss: 67.5	ND	Neoplasms, glandular and epithelial, adenocarcinoma, carcinoma
101	TRPC4	NM_016179	transient receptor potential cation channel, subfamily C, member 4	13q13.3	Loss: 65	Yes	Neoplasms, glandular and epithelial, adenocarcinoma, carcinoma
102	UBE1C*	NM_003968	ubiquitin-like modifier activating enzyme 3	3p13	Gain: 72.5	Yes	Regulation of cell cycle

103	UCN	NM_003353	urocortin	2p22.2-23.3	Loss: 62.5	Yes	Regulation of apoptosis, regulation of programmed cell death
104	VAMP1	NM_014231	vesicle-associated membrane protein 1 (synaptobrevin 1)	12p13.3	Gain: 65	Yes	Adenoma, neoplasms, glandular and epithelial, Carcinoma
105	VIPR1	NM_004624	vasoactive intestinal peptide receptor 1	3p22.1	Loss: 67.5	No	Respiratory tract neoplasm, thoracic neoplasms, neoplasms, glandular and epithelial, adenocarcinoma, lung neoplasms, carcinoma
106	VWF	NM_000552	von Willebrand factor	12p13.3	Gain: 65	No	Respiratory tract neoplasm, thoracic neoplasms, neoplasms, glandular and epithelial, adenocarcinoma, lung neoplasms, carcinoma
107	XDH*	NM_000379	xanthine dehydrogenase	2p24.3-25.1	Loss: 50	No	Aminoacid metabolism/ tyrosine metabolism, vitamin and cofactor metabolism/ retinol metabolism / regulation of cell differentiation
108	YEATS4	NM_006530	YEATS domain containing 4	12q15	Loss: 55	No	Neoplasms, glandular and epithelial
109	YWHAE	NM_006761	tyrosine 3-monooxygenase/tryptophan 5-monooxygenase activation protein, epsilon polypeptide?	17p13.3	Gain: 60	Yes	Respiratory tract neoplasm, thoracic neoplasms, neoplasms, glandular and epithelial, adenocarcinoma, lung neoplasms, carcinoma
110	ZDHHC16	NM_032327	zinc finger, DHHC domain containing 16	10q24.1	Gain: 57.5	Yes	Cell death, apoptosis
111	ZFYVE27	NM_001002261	zinc finger, FYVE domain containing 27	10q24.1	Gain: 57.5	No	Cell death
112	ZNF322A*	NM_024639	zinc finger protein 322A	6p22.1	Gain: 70	Yes	Regulation of transcription

113	ZNF35	NM_003420	zinc finger protein 35 (clone HF.10)	3p22.1-21.33	Loss: 65	Yes	Respiratory tract neoplasm, thoracic neoplasms, neoplasms, glandular and epithelial, adenocarcinoma, lung neoplasms, carcinoma / regulation of gene expression
114	ZNF384	NM_001039916	zinc finger protein 384	12p13.3	Gain: 65	Yes	Neoplasms, glandular and epithelial, carcinoma
	[a]The common candidate genes in both Asian and Caucasian are labeled with asterisk (*)				ND: non-determined		

Code	Caucasian gene name	Accession	Gene full name	Cytoband	Array CGH alteration freq.(%)	Correlation with CGAP database	Cellular pathway / Disease biomarker / Gene ontology analysis from MetaCore
1	A2M	NM_000014	alpha-2-macroglobulin	12p13.31	Loss: 50	No	Regulation of gene expression
2	ABT1	NM_013375	activator of basal transcription 1	6p22.1	Gain: 60	Yes	Regulation of gene expression
3	ARF4	NM_001660	ADP-ribosylation factor 4	3p14.3	Gain: 50	Yes	Epidermal growth factor receptor signaling pathway
4	ASPA	NM_000049	aspartoacylase (Canavan disease)	17p13.3	Gain: 65	Yes	Positive regulation of cell differentiation, regulation of cell differentiation
5	BMPR1A	NM_004329	bone morphogenetic protein receptor, type IA	10q23.2	Loss: 70	No	Adenoma / positive regulation of cell differentiation, positive regulation of cell proliferation, regulation of cell differentiation, positive regulation of epithelial cell proliferation, regulation of cell proliferation, regulation of epithelial cell proliferation
6	CAV3	NM_001234	caveolin 3	3p26.1-25.3	Gain: 50	Yes	Regulation of cell differentiation, regulation of microtubule cytoskeleton organization
7	CCNA1*	NM_003914	cyclin A1	13q13.3	Loss: 80	No	Cell cycle/ regulation of G2/S transition, cell cycle/ chromosome condensation in prometaphase / adenocarcinoma
8	CCR1	NM_001295	chemokine (C-C motif) receptor 1	3p21.31	Gain: 60	Yes	Cell migration
9	CCR4	NM_005508	chemokine (C-C motif) receptor 4	3p22.3	Loss: 75	No	Cell migration
10	CCR5	NM_000579	chemokine (C-C motif) receptor 5	3p21.31	Gain: 60	Yes	Adenocarcinoma / cell migration
11	CD2AP	NM_012120	CD2-associated protein	6p12.3	Gain: 50	Yes	Cell migration

12	CDCP1	NM_022842	CUB domain-containing protein 1	3p21.33-21.31	Gain: 60	Yes	Respiratory tract neoplasm, thoracic neoplasms, neoplasms, glandular and epithelial, adenocarcinoma, lung neoplasms, carcinoma
13	CGGBP1	NM_001008390	CGG triplet repeat binding protein 1	3p11.2-12.1	Loss: 80	Yes	Regulation of gene expression
14	CLASP2*	NM_015097	cytoplasmic linker associated protein 2	3p22.3	Loss: 80	No	Chromosome aberration / regulation of microtubule cytoskeleton organization, cell migration
15	CLEC3B	NM_003278	C-type lectin domain family 3, member B	3p21.33-21.31	Gain: 60	Yes	Adenocarcinoma
16	CNTN4	NM_175607	contactin 4	3p26.3	Gain: 55	Yes	Regulation of cell differentiation
17	DCLK3	NM_033403	doublecortin-like kinase 3	3p22.2	Loss: 65	Yes	Carcinoma, large cell, adenocarcinoma, carcinoma, non-small-cell lung
18	DDX1	NM_004939	DEAD (Asp-Glu-Ala-Asp) box polypeptide 1	2p24.3	Gain: 55	Yes	Regulation of gene expression
19	DDX53	NM_182699	DEAD (Asp-Glu-Ala-Asp) box polypeptide 53	Xp22.11	Gain: 60	Yes	Adenocarcinoma
20	EDEM1	NM_014674	ER degradation enhancer, mannosidase alpha-like 1	3p26.2-26.1	Gain: 50	Yes	Apoptosis and survival/ endoplasmic reticulum stress response pathway
21	EIF4E3	NM_173359	eukaryotic translation initiation factor 4E family member 3	3p13-14.1	Gain: 70	Yes	Regulation of gene expression
22	ESD	NM_001984	esterase D/formylglutathione hydrolase	13q14.13-14.2	Gain: 70	Yes	Adenocarcinoma
23	FAM107A	NM_007177	family with sequence similarity 107, member A	3p14.3-14.2	Gain: 50	Yes	Adenocarcinoma

24	FHIT	NM_002012	fragile histidine triad gene	3p14.3-14.2	Gain: 50	No	Chromosome aberrations, carcinoma, large cell, adenocarcinoma, adenoma, carcinoma, non-small-cell lung
25	FOXJ2	NM_018416	forkhead box J2	12p13.31	Loss: 50	Yes	Regulation of gene expression
26	FOXP1*	NM_032682	forkhead box P1	3p13-14.1	Gain: 70	Yes	Chromosome aberration, neoplasms, glandular and epithelial, adenocarcinoma, carcinoma / positive regulation of epithelial cell proliferation
27	GDF7	NM_182828	growth differentiation factor 7	2p24.1	Gain: 55	Yes	Regulation of gene expression
28	GLUD1	NM_005271	glutamate dehydrogenase 1	10q23.2	Loss: 70	No	Aminoacid metabolism/ proline metabolism, aminoacid metabolism/ urea cycle
29	HDGFL1	NM_138574	hepatoma derived growth factor-like 1	6p22.3	Gain: 50	No	Adenocarcinoma
30	HESX1	NM_003865	homeo box (expressed in ES cells) 1	3p14.3	Gain: 50	Yes	Regulation of gene expression
31	HIST1H1A	NM_005325	histone 1, H1a	6p22.1	Gain: 60	Yes	Cell cycle/ Chromosome condensation in prometaphase
32	HIST1H1C	NM_005319	histone 1, H1c	6p22.1	Gain: 50	Yes	Cell cycle/ Chromosome condensation in prometaphase
33	HIST1H1D	NM_005320	histone 1, H1d	6p22.1	Gain: 50	Yes	Cell cycle/ Chromosome condensation in prometaphase
34	HIST1H1E	NM_005321	histone 1, H1e	6p22.1	Gain: 50	Yes	Cell cycle/ Chromosome condensation in prometaphase
35	HIST1H1T	NM_005323	histone 1, H1t	6p22.1	Gain: 50	Yes	Cell cycle/ Chromosome condensation in prometaphase
36	HIST1H4A	NM_003538	histone 1, H4a	6p22.1	Gain: 60	No	Transcription factors regulatory processes/ NOTCH3-mediated pathway for NF-KB activity modulation
37	HIST1H4B	NM_003544	histone 1, H4b	6p22.1	Gain: 60	Yes	Transcription factors regulatory processes/ NOTCH4-mediated pathway for NF-KB activity modulation

38	HIST1H4C	NM_003542	histone 1, H4c	6p22.1	Gain: 50	No	Transcription factors regulatory processes/ NOTCH5-mediated pathway for NF-KB activity modulation
39	HIST1H4D	NM_003539	histone 1, H4d	6p22.1	Gain: 50	No	Transcription factors regulatory processes/ NOTCH6-mediated pathway for NF-KB activity modulation
40	HIST1H4E	NM_003545	histone 1, H4e	6p22.1	Gain: 50	No	Transcription factors regulatory processes/ NOTCH7-mediated pathway for NF-KB activity modulation
41	HIST1H4F	NM_003540	histone 1, H4f	6p22.1	Gain: 50	Yes	Transcription factors regulatory processes/ NOTCH8-mediated pathway for NF-KB activity modulation
42	HIST1H4G	NM_003547	histone 1, H4g	6p22.1	Gain: 60	No	Transcription factors regulatory processes/ NOTCH9-mediated pathway for NF-KB activity modulation
43	HIST1H4H	NM_003543	histone 1, H4h	6p22.1	Gain: 60	No	Transcription factors regulatory processes/ NOTCH10-mediated pathway for NF-KB activity modulation
44	HIST1H4I	NM_003495	histone 1, H4i	6p22.1	Gain: 50	No	Transcription factors regulatory processes/ NOTCH11-mediated pathway for NF-KB activity modulation
45	ITPR1	NM_002222	inositol 1,4,5-triphosphate receptor, type 1	3p26.3-26.2	Gain: 55	No	Angiogenesis/ Cross-talk between VEGF and Angiopoietin 2 signaling pathways, apoptosis and survival/ endoplasmic, reticulum stress response pathway
46	LIMD1	NM_014240	LIM domains containing 1	3p21.31	Gain: 60	No	Regulation of gene expression
47	LMCD1	NM_014583	LIM and cysteine-rich domains 1	3p26.1	Gain: 50	Yes	Regulation of gene expression, negative regulation of gene expression
48	LTF	NM_002343	lactotransferrin	3p21.31	Gain: 60	Yes	Carcinoma, non-small-cell lung

49	MBTPS2	NM_015884	membrane-bound transcription factor protease, site 2	Xp22.11	Gain: 60	Yes	Apoptosis and survival/ Endoplasmic reticulum stress response pathway, Regulation of lipid metabolism/ Regulation of fatty acid synthase activity in hepatocytes
50	MDGA1	NM_153487	MAM domain containing glycosylphosphatidylinositol anchor 1	6p21.2	Loss: 85	Yes	Cell migration
51	MITF*	NM_198159	microphthalmia-associated transcription factor	3p14.1	Gain: 70	Yes	Neoplasms, glandular and epithelial, Carcinoma / regulation of cell differentiation, regulation of cell proliferation, regulation of gene expression
52	MSGN1	NM_001105569	mesogenin 1	2p24.2	Gain: 55	ND	Regulation of gene expression
53	MYCN	NM_005378	v-myc myelocytomatosis viral related oncogene, neuroblastoma derived (avian)	2p24.3	Gain: 55	Yes	Chromosome Aberrations / positive regulation of cell proliferation, regulation of cell proliferation, regulation of gene expression
54	NT5C1B	NM_001002006	5'-nucleotidase, cytosolic IB	2p24.2	Gain: 55	Yes	Nucleotide metabolism/ dCTP/dUTP; dATP/dITP metabolism
55	NTSR2	NM_012344	neurotensin receptor 2	2q25.1	Gain: 50	Yes	Adenoma
56	NUFIP1	NM_012345	nuclear fragile X mental retardation protein interacting protein 1	13q14.11-14.12	Gain: 80	Yes	Regulation of gene expression
57	PAFAH1B1*	NM_015345	platelet-activating factor acetylhydrolase, isoform Ib, alpha subunit 45kDa	17p13.3	Gain: 70	Yes	Cytoskeleton organization
58	PGBD1	NM_032507	piggyBac transposable element derived 1	6p22.1	Gain: 55	Yes	Regulation of gene expression

59	POLA1	NM_016937	polymerase (DNA directed), alpha 1, catalytic subunit	Xp22.11	Gain: 60	Yes	Nucleotide metabolism/ dCTP/dUTP; dATP/dITP metabolism
60	POU1F1	NM_000306	POU class 1 homeobox 1	3p11.2-12.1	Loss: 70	No	Adenoma / positive regulation of cell proliferation, regulation of cell proliferation, regulation of gene expression
61	PRL	NM_000948	prolactin	6p22.3	Gain: 50	Yes	Positive regulation of cell proliferation, positive regulation of epithelial cell proliferation, regulation of cell proliferation, regulation of epithelial cell proliferation
62	PROK2	NM_021935	prokineticin 2	3p13-14.1	Gain: 70	Yes	Angiogenesis
63	PUM2	NM_015317	pumilio homolog 2 (Drosophila)	2p24.1	Gain: 55	Yes	Regulation of gene expression
64	RAD18	NM_020165	RAD18 homolog (S. cerevisiae)	3p26.1-25.3	Gain: 50	Yes	Carcinoma, non-small-cell lung
65	RBBP7	NM_002893	retinoblastoma binding protein 7	Xp22.13-22.2	Gain: 50	Yes	Transcription factors regulatory processes/ NOTCH13-mediated pathway for NF-KB activity modulation / regulation of gene expression, negative regulation of gene expression
66	REPS2	NM_001080975	RALBP1 associated Eps domain containing 2	Xp22.13-22.2	Gain: 50	Yes	Epidermal growth factor receptor signaling pathway
67	RhoB	NM_004040	ras homolog gene family, member B	2p24.1	Gain: 55	Yes	Angiogenesis
68	ROBO1	NM_133631	roundabout, axon guidance receptor, homolog 1 (Drosophila)	3p12.3	Gain: 65	Yes	Positive regulation of cell differentiation, regulation of cell differentiation
69	ROBO2	NM_002942	roundabout, axon guidance receptor, homolog 2 (Drosophila)	3p12.3	Gain: 70	Yes	Positive regulation of cell differentiation, regulation of cell differentiation

70	RYBP*	NM_012234	RING1 and YY1 binding protein	3p13-14.1	Gain: 70	Yes	Cell death, apoptosis
71	SCML1	NM_006746	sex comb on midleg-like 1 (Drosophila)	Xp22.13	Gain: 50	Yes	Regulation of gene expression
72	SCML2	NM_006089	sex comb on midleg-like 2 (Drosophila)	Xp22.13	Gain: 60	Yes	Regulation of gene expression
73	SMAD9	NM_005905	SMAD family member 9	13q13.3	Loss: 55	No	Positive regulation of cell differentiation, regulation of cell differentiation, regulation of gene expression
74	SMS	NM_004595	spermine synthase	Xp22.11	Gain: 60	No	Amino acid metabolism/ urea cycle
75	SNCG	NM_003087	synuclein, gamma (breast cancer-specific protein 1)	10q23.2	Loss: 70	Yes	Adenocarcinoma
76	SNRK	NM_001100594	SNF related kinase	3p22.1-21.33	Gain: 60	Yes	Neoplasms, glandular and epithelial, Adenocarcinoma, Carcinoma / regulation of apoptosis, regulation of programmed cell death
77	SOX4	NM_003107	SRY (sex determining region Y)-box 4	6p22.3	Gain: 70	Yes	Respiratory tract neoplasm, thoracic neoplasms, neoplasms, glandular and epithelial, adenocarcinoma, lung neoplasms, carcinoma / regulation of apoptosis, negative regulation of apoptosis, regulation of programmed cell death, negative regulation of programmed cell death, negative regulation of cell death
78	SS18L2	NM_016305	synovial sarcoma translocation gene on chromosome 18-like 2	3p22.1	Gain: 55	Yes	Chromosome aberration, neoplasms, glandular and epithelial, Adenocarcinoma, Carcinoma
79	TMF1	NM_007114	TATA element modulatory factor 1	3p14.1	Gain: 70	Yes	Regulation of gene expression

80	TNFSF11	NM_033012	tumor necrosis factor (ligand) superfamily, member 11	13q14.11	Gain: 80	Yes	Apoptosis and survival/ Anti-apoptotic TNFs/NF-kB/Bcl-4 pathway / adenocarcinoma / positive regulation of cell differentiation, epithelial cell proliferation, regulation of cell differentiation, regulation of I-kappaB kinase/NF-kappaB cascade
81	TRPM6	NM_017662	transient receptor potential cation channel, subfamily M, member 6	9q21.13	Gain: 60	Yes	Carcinoma, large cell
82	TSC22D1	NM_006022	TSC22 domain family, member 1	13q14.11-14.12	Gain: 90	Yes	Regulation of gene expression
83	UBE1C*	NM_003968	ubiquitin-like modifier activating enzyme	3p14.1	Gain: 70	Yes	Regulation of cell cycle
84	UBP1	NM_014517	upstream binding protein 1 (LBP-1a)	3p22.3	Loss: 80	Yes	Regulation of gene expression, negative regulation of gene expression, angiogenesis
85	VGLL3	NM_016206	vestigial like 3 (Drosophila)	3p11.2-12.1	Loss: 70	Yes	Regulation of gene expression
86	VSNL1	NM_003385	visinin-like 1	2p24.2	Gain: 55	ND	Carcinoma, non-small-cell lung
87	WAPAL	NM_015045	wings apart-like homolog (Drosophila)	10q23.2	Loss: 70	Yes	Chromosome aberrations
88	XDH*	NM_000379	xanthine dehydrogenase	2p24.3-25.1	Loss: 50	Yes	Regulation of cell differentiation
89	YY2	NM_206923	YY2 transcription factor	Xp22.11	Gain: 60	Yes	Regulation of gene expression
90	ZBTB47	NM_145166	zinc finger and BTB domain containing 47	3p22.1	Gain: 55	Yes	Regulation of gene expression
91	ZFX	NM_003410	zinc finger protein, X-linked	Xp22.11	Gain: 50	Yes	Regulation of gene expression
92	ZNF165	NM_003447	zinc finger protein 165	6p22.1	Gain: 55	Yes	Regulation of gene expression
93	ZNF167	NM_018651	zinc finger protein 167	3p21.33-21.31	Gain: 60	Yes	Regulation of gene expression

94	ZNF184	NM_007149	zinc finger protein 184 (Kruppel-like)	6p22.1	Gain: 50	Yes	Regulation of gene expression
95	ZNF187	NM_001023560	zinc finger protein 187	6p22.1	Gain: 55	Yes	Regulation of gene expression
96	ZNF192	NM_006298	zinc finger protein 192	6p22.1	Gain: 55	No	Regulation of gene expression
97	ZNF193	NM_006299	zinc finger protein 193	6p22.1	Gain: 55	Yes	Regulation of gene expression
98	ZNF197	NM_006991	zinc finger protein 197	3p21.33-21.31	Gain: 60	Yes	Regulation of gene expression
99	ZNF311	NM_001010877	zinc finger protein 311	6p22.1	Loss: 55	No	Regulation of gene expression
100	ZNF322A*	NM_024639	zinc finger protein 322A	6p22.1	Gain: 50	Yes	Regulation of transcription
101	ZNF35	NM_003420	zinc finger protein 35 (clone HF.10)	3p21.33-21.31	Gain: 60	Yes	Respiratory tract neoplasm, thoracic neoplasms, neoplasms, glandular and epithelial, adenocarcinoma, lung neoplasms, carcinoma / regulation of gene expression
102	ZNF391	NM_001076781	zinc finger protein 391	6p22.1	Gain: 50	Yes	Regulation of gene expression
103	ZNF445	NM_181489	zinc finger protein 445	3p21.33-21.31	Gain: 60	Yes	Regulation of gene expression
104	ZNF452	NM_052923	zinc finger protein 452	6p22.1	Gain: 50	Yes	Regulation of gene expression
105	ZNF501	NM_145044	zinc finger protein 501	3p21.33-21.31	Gain: 60	Yes	Regulation of gene expression
106	ZNF502	NM_033210	zinc finger protein 502	3p21.33-21.31	Gain: 60	Yes	Regulation of gene expression
107	ZNF660	NM_173658	zinc finger protein 660	3p21.33-21.31	Gain: 60	Yes	Regulation of gene expression
108	ZNF662	NM_207404	zinc finger protein 662	3p22.1-21.33	Gain: 60	Yes	Regulation of gene expression
109	ZNF705A	NM_001004328	zinc finger protein 705A	12p13.31	Loss: 50	No	Regulation of gene expression
	[a]The common candidate genes in both Asian and Caucasian are labeled with asterisk (*)				ND: non-determined		
