# Supplementary material for: The database of chromosome imbalance regions and genes resided in lung cancer from Asian and Caucasian identified by array-comparative genomic hybridization
Source: BMC Cancer. 2012 Jun 12;12:235. doi: 10.1186/1471-2407-12-235 (PMC3488578; doi:10.1186/1471-2407-12-235)
Supplement: Additional file 6 — Table S5.Association between mRNA expression level and lung cancer clinicopathological parameters of four candidate genes in Asian. [file 1471-2407-12-235-S6.pdf]

Table S5. Association between mRNA expression level and lung cancer clinicopathological parameters of four candidate genes in Asian [a].

Characteristics		Total [b]	*ARHGAP19* overexpression [c]		*ARHGAP19* Normal		*FRAT2* overexpression [c]		*FRAT2* Normal		*PAFAH1B1* overexpression [c]		*PAFAH1B1* Normal		*ZNF322A* overexpression [c]		*ZNF322A* Normal	
		n	n	(%)	n	(%)	n	(%)	n	(%)	n	(%)	n	(%)	n	(%)	n	(%)
Overall		45	33	(73.3)	12	(26.7)	23	(51.1)	22	(48.9)	31	(68.9)	14	(31.1)	27	(60.0)	18	(40.0)
Clinicopathological parameters																		
Age	＜65	22	13	(59.1)	9	(40.9)	10	(45.5)	12	(54.5)	15	(68.2)	7	(31.1)	14	(63.6)	8	(36.4)
	≧65	23	20	(87.9) [0.035]	3	(13.1)	13	(56.5)	10	(43.5)	16	(69.6)	7	(30.4)	13	(56.5)	10	(43.5)
Sex	Male	36	29	(80.6) [0.028]	37	(19.4)	18	(50.0)	18	(50.0)	25	(69.4)	11	(30.6)	18	(50)	18	(50)
	Female	9	4	(44.4)	10	(55.6)	5	(55.6)	4	(44.4)	6	(66.7)	3	(33.3)	5	(55.6)	4	(44.4)
Smoker	Yes	18	15	(83.3) [0.019]	3	(16.7)	7	(38.8)	11	(61.2)	14	(77.8)	4	(22.2)	11	(61.1)	7	(38.9)
	No	8	3	(37.5)	5	(62.5)	5	(62.5)	3	(37.5)	5	(62.5)	3	(37.5)	6	(75.0)	2	(25.0)
Tumor type																		
ADC		28	16	(57.1)	12	(42.9)	10	(35.7)	18	(64.3)	18	(64.3)	10	(35.7)	15	(53.6)	13	(46.4)
SCC		17	17	(100) [0.002]	0	(0)	13	(76.5) [0.008]	4	(23.5)	13	(76.5)	4	(23.5)	12	(70.6)	5	(29.4)
Tumor stage																		
I + II		21	16	(76.2)	5	(23.8)	10	(52.4)	11	(47.6)	13	(59.1)	9	(40.9)	14	(66.7)	7	(33.3)
III + IV		23	16	(69.6)	7	(30.4)	12	(52.2)	11	(47.8)	18	(78.3)	5	(21.7)	12	(52.2)	11	(47.8)
Correlation with protein		28																
IHC	Over expression										14	(73.7) [0.041]	5	(26.3)	16	(84) [0.030]	3	(16)
	Normal expression										3	(33.3)	6	(66.7)	4	(44.4)	5	(55.6)

[a] The *P* values with significance are shown as superscripts.

[b] Total number of samples in some categories is less than the overall number analyzed because clinical data or molecular data was not available for these samples.

[c] The mRNA overexpression of candidate genes was defined using the mean mRNA expression of normal lung tissue as a cutoff value to determine patients with mRNA overexpression.
